# Frequency and mortality of septic shock in Europe and North America: a systematic review and meta-analysis

**DOI:** 10.1186/s13054-019-2478-6

**Published:** 2019-05-31

**Authors:** Jean-Louis Vincent, Gabriel Jones, Sholto David, Elena Olariu, Kevin K. Cadwell

**Affiliations:** 1 Department of Intensive Care, Erasme Hospital, Université libre de Bruxelles, Route de Lennik 808, 1070 Brussels, Belgium; 2 PHMR Ltd., Berkeley Works, Berkley Grove, London, NW1 8XY UK

**Keywords:** Sepsis, Intensive care, Heterogeneity, Outcome, Sepsis definition

## Abstract

**Background:**

Septic shock is the most severe form of sepsis, in which profound underlying abnormalities in circulatory and cellular/metabolic parameters lead to substantially increased mortality. A clear understanding and up-to-date assessment of the burden and epidemiology of septic shock are needed to help guide resource allocation and thus ultimately improve patient care. The aim of this systematic review and meta-analysis was therefore to provide a recent evaluation of the frequency of septic shock in intensive care units (ICUs) and associated ICU and hospital mortality.

**Methods:**

We searched MEDLINE, Embase, and the Cochrane Library from 1 January 2005 to 20 February 2018 for observational studies that reported on the frequency and mortality of septic shock. Four reviewers independently selected studies and extracted data. Disagreements were resolved via consensus. Random effects meta-analyses were performed to estimate pooled frequency of septic shock diagnosed at admission and during the ICU stay and to estimate septic shock mortality in the ICU, hospital, and at 28 or 30 days.

**Results:**

The literature search identified 6291 records of which 71 articles met the inclusion criteria. The frequency of septic shock was estimated at 10.4% (95% CI 5.9 to 16.1%) in studies reporting values for patients diagnosed at ICU admission and at 8.3% (95% CI 6.1 to 10.7%) in studies reporting values for patients diagnosed at any time during the ICU stay. ICU mortality was 37.3% (95% CI 31.5 to 43.5%), hospital mortality 39.0% (95% CI 34.4 to 43.9%), and 28-/30-day mortality 36.7% (95% CI 32.8 to 40.8%). Significant between-study heterogeneity was observed.

**Conclusions:**

Our literature review reaffirms the continued common occurrence of septic shock and estimates a high mortality of around 38%. The high level of heterogeneity observed in this review may be driven by variability in defining and applying the diagnostic criteria, as well as differences in treatment and care across settings and countries.

**Electronic supplementary material:**

The online version of this article (10.1186/s13054-019-2478-6) contains supplementary material, which is available to authorized users.

## Introduction

Sepsis, the association of organ dysfunction with an infection, is a complex multifactorial disease that has severe health and economic burden on both the patient and healthcare systems worldwide. Sepsis is one of the leading causes of death and critical illness in the world [1, 2], with in-hospital mortality rates in the USA as high as 25–30% [3]. Data drawn principally from large retrospective database studies point to high population-level incidence rates for sepsis among patients hospitalized in high-income countries; indeed, admissions of patients with sepsis exceed those of patients who have suffered myocardial infarction or stroke [4, 5]. The incidence of sepsis in developed countries has been reported to be increasing [6, 7], although this is controversial and may in large part be a phenomenon of reporting bias related to financial reimbursement, increased awareness of sepsis definitions among medical professionals, and changes in diagnostic codes [8–11]. A recent study in which sepsis was defined according to the latest guidelines suggested relative stability in the rate of sepsis from 2002 to 2012 [12].

Septic shock, characterized by arterial hypotension, altered tissue perfusion, and increased blood lactate levels [13, 14], is the most severe form of sepsis. In 2016, Shankar-Hari et al. [15] conducted a systematic review to evaluate clinical criteria currently used to identify septic shock. They reported worldwide estimates for hospital mortality but provided no information on intensive care unit (ICU) or 28-/30-day mortality rates or on the frequency of septic shock. Moreover, Shankar-Hari et al. included data from patients enrolled between 1989 and 2015 (publications from 1992 to 2015), yet our awareness of sepsis has increased considerably since then and patient management has also changed.

We therefore conducted an updated systematic review to identify observational studies conducted in Europe and North America that reported epidemiological data of patients with septic shock. This was followed by a quantitative meta-analysis to determine the frequency of septic shock and its associated mortality.

## Materials and methods

A systematic review protocol was prepared based on the Preferred Reporting Items for Systematic Review and Meta-Analysis Protocols (PRISMA-P) [16] and in accordance with established guidelines from The Cochrane Handbook for Systematic Reviews of Interventions [17] and the Center for Reviews and Dissemination [18].

### Search strategy

A comprehensive literature search strategy combining Medical Subject Headings (MeSH) and free text terms for the epidemiology of septic shock, health-related quality of life, costs, and treatment guidelines was used to retrieve articles of interest from MEDLINE, MEDLINE In-Process Citations & Daily Update, Embase, and the Cochrane Library databases. Searches were limited to papers published between 1 January 2005 and 20 February 2018. Full search strategies are provided in Additional file 1.

### Study selection criteria

Observational studies of adult patients (aged ≥ 15 years) with sepsis were eligible for inclusion if they reported data on either frequency or mortality of septic shock. Studies were limited to those conducted in Europe, the USA, or Canada. For the frequency assessment, they must have included cohorts of ≥ 100 patients, and for the mortality assessment, cohorts of ≥ 15 patients. Non-observational studies or studies of patients within specific disease groups or other exclusive populations were excluded. Conference abstracts, reviews, systematic reviews, and studies indexed as case reports, editorials, and letters were also excluded. The full list of inclusion and exclusion criteria is available in Additional file 1.

### Screening and study selection

Double screening was performed, with four authors (K.K.C., E.O., S.D., and G.J.) in pairs providing an independent assessment of the titles and abstracts of all records retrieved in the electronic searches. The full text of each study that met the criteria for inclusion was then reviewed, with discrepancies between the reviewers resolved through discussion.

### Data extraction

The following data were extracted from papers identified at full-text screening: article identifiers (authors, year of publication, objective), study identifiers (sample size, design, country, length of follow-up, inclusion criteria, definition and criteria used for identifying septic shock, comorbidities, number of organ failures), setting and population (age, sex, reason for admission, severity scores, acquisition of infection, microorganisms identified), and outcome measures (frequency, incidence, mortality).

### Assessment of methodological quality

Quality assessments of the studies that were eligible for meta-analysis were performed using “The Joanna Briggs Institute Critical Appraisal Checklist for studies reporting prevalence data” [19] for frequency studies, with a modified version of “The Joanna Briggs Institute Critical Appraisal Checklist for case series” [20] used for studies reporting mortality.

### Statistical analysis and meta-analyses

Random effects meta-analyses were performed to estimate pooled frequencies of septic shock diagnosed at admission or during the ICU stay and to estimate septic shock mortality in the ICU, hospital, and at 28 or 30 days. Separate analyses were performed on studies in which The Third International Consensus Definitions for Sepsis and Septic Shock (Sepsis-3) [14] were used, as this introduced hyperlactatemia as a component for septic shock. Using frequency and mortality rates from patients diagnosed with Sepsis-3 definitions was considered a potential source of heterogeneity given the more narrowly defined, more severe patients these criteria would identify with the addition of this component. Unless otherwise stated, estimates given are derived from studies in which any criteria except Sepsis-3 are used to identify septic shock. Confidence intervals for individual studies were calculated using the Clopper-Pearson method and between-study variances were estimated using the DerSimonian-Laird technique. For frequency, data were pooled using the inverse-variance method and the Freeman-Tukey double arcsine transformation to calculate an overall proportion, before back transformation to the original scale and the logit transformation were used to pool hospital mortality data. A continuity correction of 0.5 was applied in studies with zero cell frequencies. Statistical heterogeneity was assessed visually using forest plots and formally using the *I*^2^ statistic; heterogeneity was considered to be high for *I*^2^ values greater than 75% [21].

For frequency, two meta-analyses were performed according to the time of septic shock diagnosis reported in the study (at ICU admission or at any time during the ICU stay). For mortality, three meta-analyses were performed according to the type of reported mortality: ICU mortality, in-hospital mortality, and mortality at either 28 or 30 days. Subgroup meta-analyses for frequency and mortality were performed for European vs North American studies, and for single center vs multicenter studies. Publications that did not report data as proportions (*n*/*N*) but as percentages were excluded if the proportions could not be accurately back-calculated. Studies that reported 1-day point prevalence estimates were also excluded from the meta-analyses.

All analyses were performed using R software (www.R-project.org) and the package “meta” (general package for meta-analysis) (https://cran.r-project.org/web/packages/meta/meta.pdf).

## Results

### Study selection

The literature search identified 6291 records following de-duplication. Following title and abstract screening 460 articles met the criteria for full-text evaluation. A total of 71 met the eligibility criteria and were included in the qualitative systematic review; 50 publications reported data from Europe and 21 publications reported data from North America. Full details of study selection and exclusions are shown in the PRISMA flow diagram in Additional file 1: Figure S1.

Frequency data were reported in 36 of the 71 publications covering 27 individual studies/datasets [22–57] (Additional file 1: Table S1). Data from the Sepsis Occurrence in Acutely Ill Patients (SOAP) study were reported in four separate publications [26–28, 32]; data from a single 1-day frequency study from Germany were reported in two publications [29, 34]; the French EPIdemiology of Septic Shock study (EPISS) was detailed in two publications [46, 47]; a multicenter study from the Piedmont Intensive Care Unit Network in Italy was reported in two publications [48, 49]; the OUTCOMEREA database was used for three publications [22, 36, 51]; and the Portuguese Community-Acquired Sepsis (SACiUCI) study was reported in two publications [37, 39]. Mortality data were reported in 62 publications covering 57 individual studies [10, 23–28, 31, 32, 34, 35, 37–43, 45–48, 50–52, 54, 55, 57–91] (Additional file 1: Table S2).

### Quality assessment

The proportion of studies that fulfilled each item on the quality assurance checklist is shown in Additional file 1: Tables S3 and S4 for the studies that report frequency data and mortality data, respectively. For frequency, the areas that raised concern were the validity of methods used for identification of septic shock, the reliability of septic shock diagnosis among patients, and rate of response and methods employed to manage low response rate. For mortality, the areas that raised concern were, again, the reliability of septic shock diagnosis among patients, in addition to completeness of inclusion, and clear reporting of demographics of the septic shock patients.

### Frequency estimates

**Fig. 1:**
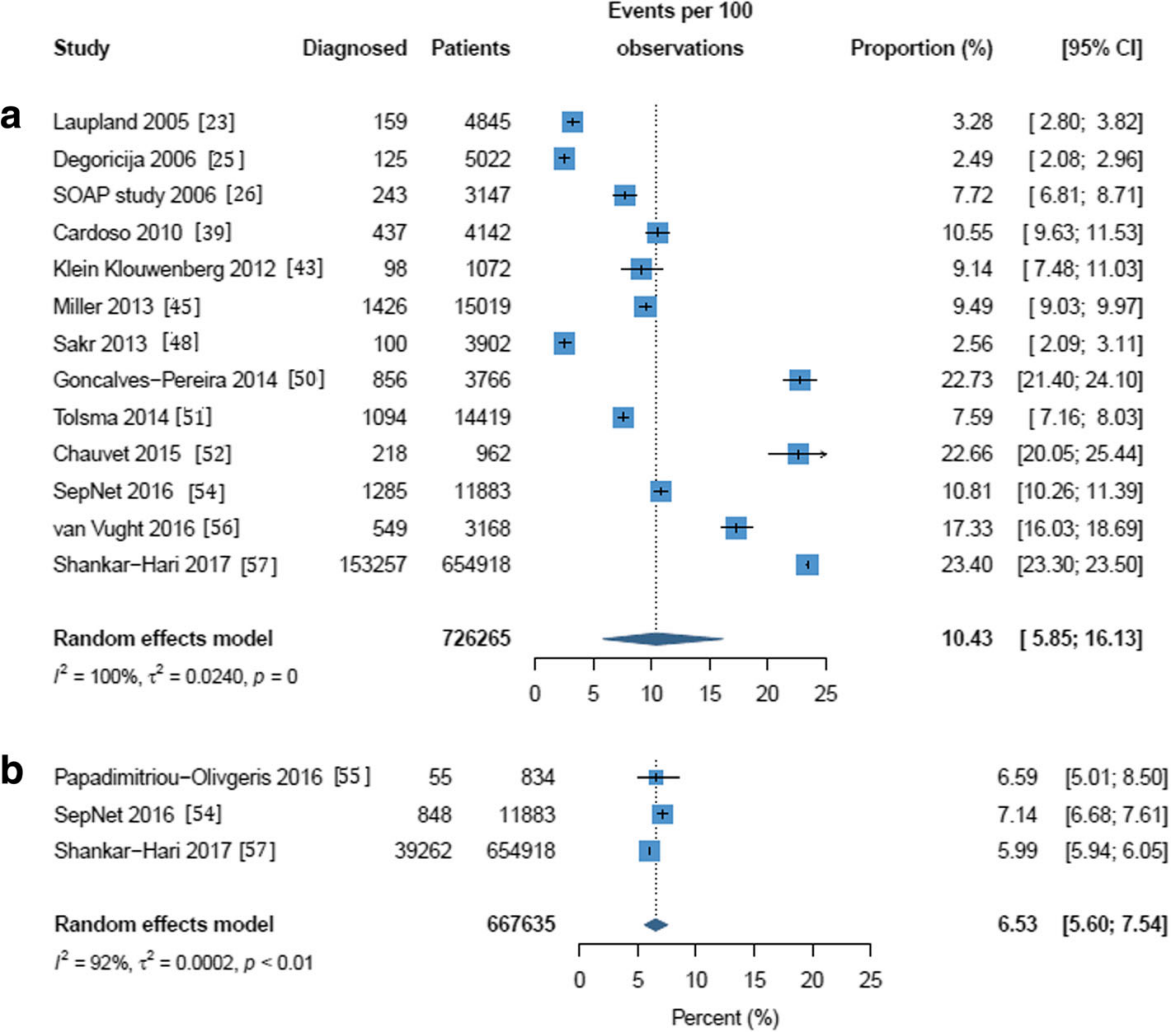
Frequency of septic shock in a cohort of patients admitted to the intensive care unit and diagnosed on admission. (a) All definitions except Sepsis-3. (b) Sepsis-3 only. The forest plots contain exact 95% confidence intervals, and specific studies are weighted using the inverse-variance method. The pooled summaries are obtained using the Freeman–Tukey double arcsine transformation and the DerSimonian–Laird method estimates between-study variance

**Fig. 2:**
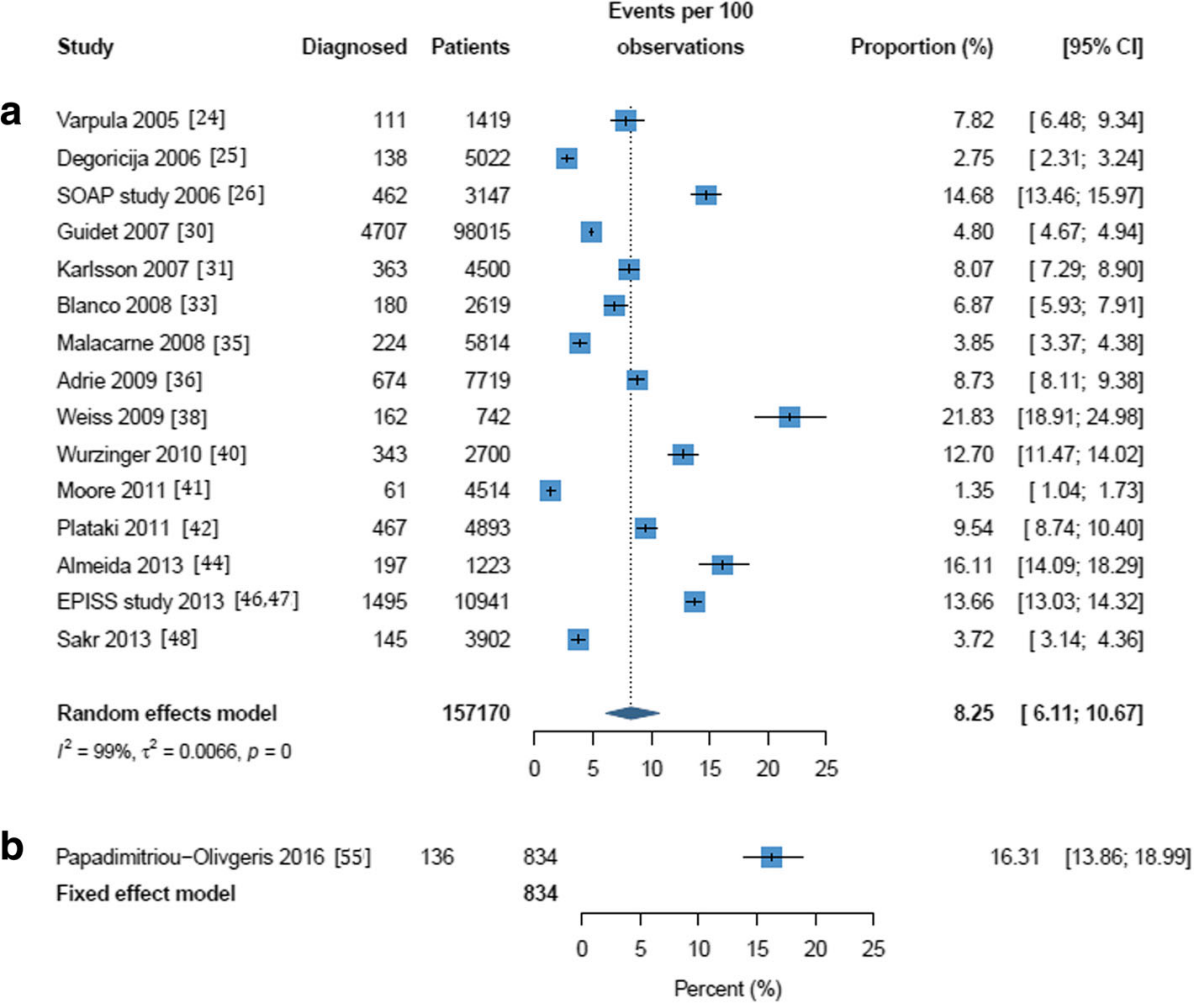
Frequency of septic shock within a cohort of patients admitted to the intensive care unit and diagnosed at some time during stay. (a) All definitions except Sepsis-3. (b) Sepsis-3 only. The forest plots contain exact 95% confidence intervals, and specific studies are weighted using the inverse-variance method. The pooled summaries are obtained using the Freeman–Tukey double arcsine transformation and the DerSimonian–Laird method estimates between-study variance

Frequency estimates ranged from 2.5% [25] to 23.4% [57] when septic shock was diagnosed at ICU admission (Additional file 1: Table S1). Three studies reported 1-day point prevalence estimates for frequency and were excluded from the meta-analyses [29, 34, 53]. The overall pooled frequency of septic shock was estimated at 10.4% (95% CI 5.9 to 16.1%) with a high level of heterogeneity (*I*^2^ = 100%; *p* = 0) (Fig. 1). This pooled frequency decreased to 6.5% (95% CI 5.6 to 7.5%) when using Sepsis-3 criteria. When septic shock was diagnosed at any time during the ICU stay, estimates ranged from 1.4% [41] to 27.6% [38] (Additional file 1: Table S1) and the overall pooled frequency of septic shock was estimated at 8.3% (95% CI 6.1 to 10.7%) with a high level of heterogeneity (*I*^2^ = 99%; *p* = 0) (Fig. 2). Estimates for septic shock frequency at admission and at any time during the ICU stay were higher in European populations than in North American populations (11.4% vs. 6.0% and 8.9% vs. 4.6% respectively; see Additional file 1: Figures S2 and S3). Estimates for septic shock frequency at admission were higher in multicenter than in single-center studies (10.6% vs. 9.0%), but the reverse was true for septic shock frequency during the ICU stay (7.6% vs. 9.9%); none of these differences were statistically significant (Additional file 1: Figures S4 and S5).

### Mortality

**Fig. 3:**
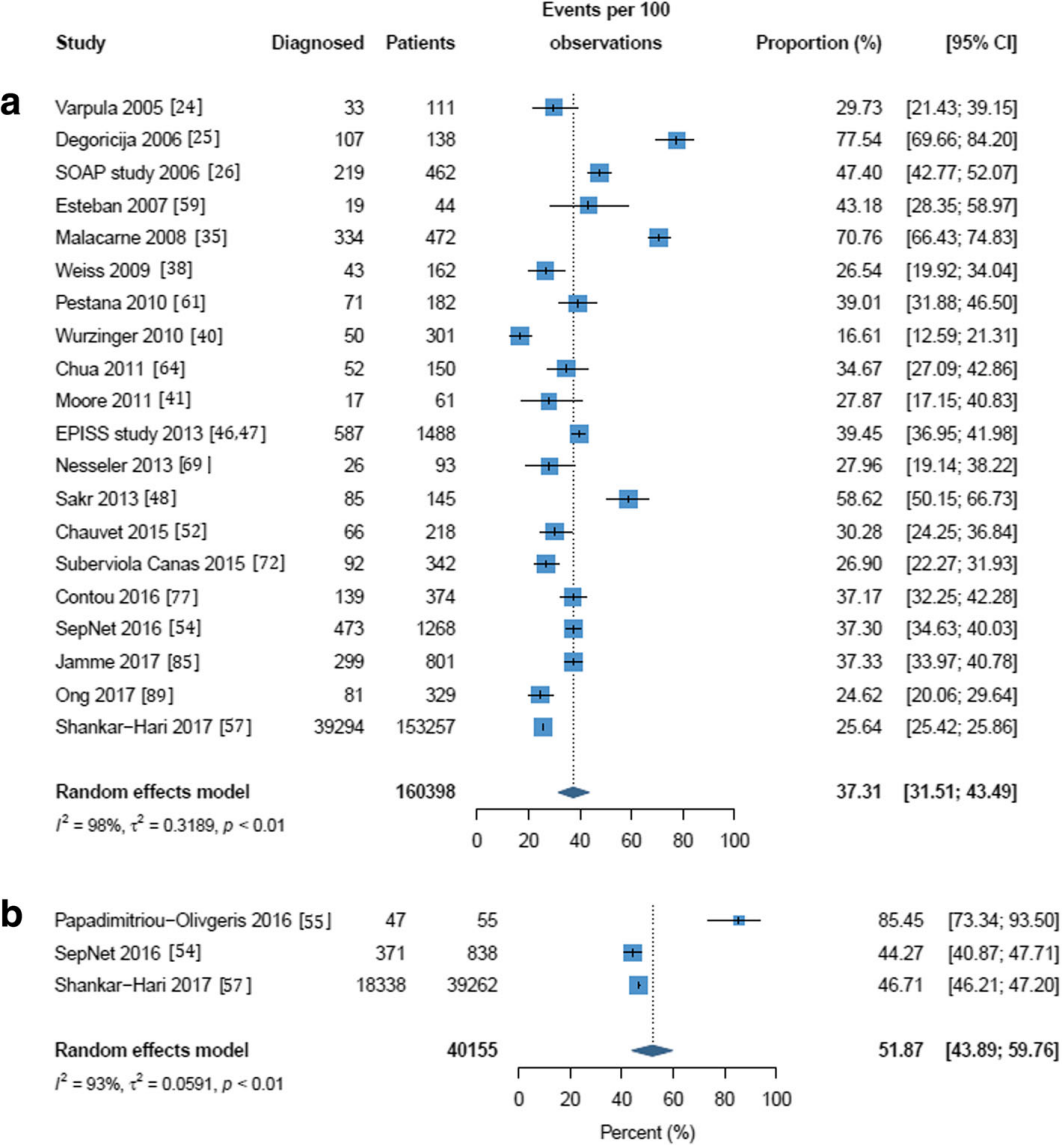
Random effects meta-analysis of studies reporting intensive care unit mortality of septic shock patients. (a) All definitions except Sepsis-3. (b) Sepsis-3 only. The forest plots contain exact 95% confidence intervals, and specific studies are weighted using the inverse-variance method. The pooled summaries are obtained using the logit transformation and the DerSimonian–Laird method estimates between-study variance

**Fig. 4:**
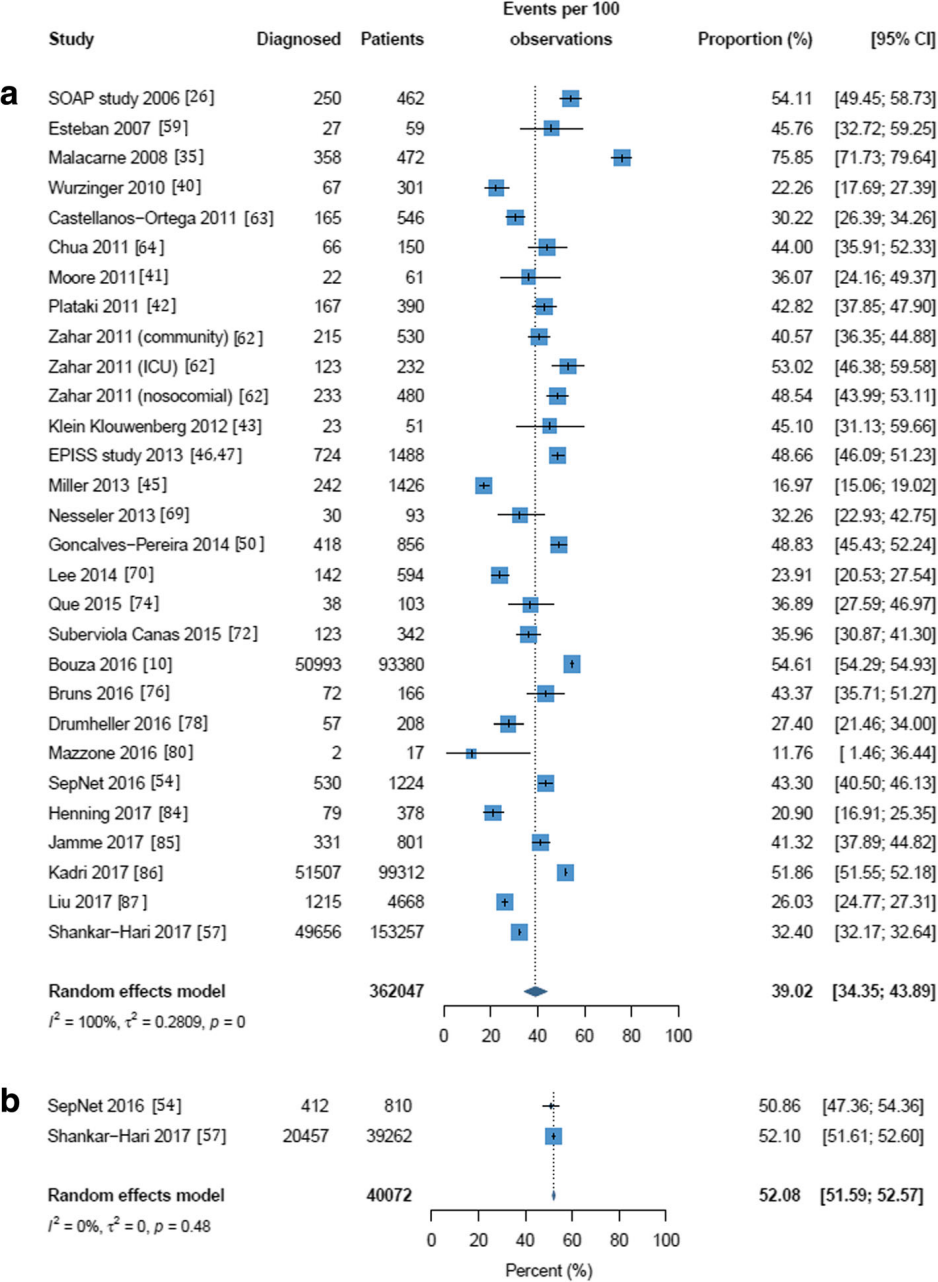
Random effects meta-analysis of studies reporting hospital mortality of septic shock patients. (a) All definitions except Sepsis-3. (b) Sepsis-3 only. The forest plots contain exact 95% confidence intervals, and specific studies are weighted using the inverse-variance method. The pooled summaries are obtained using the logit transformation and the DerSimonian–Laird method estimates between-study variance

**Fig. 5:**
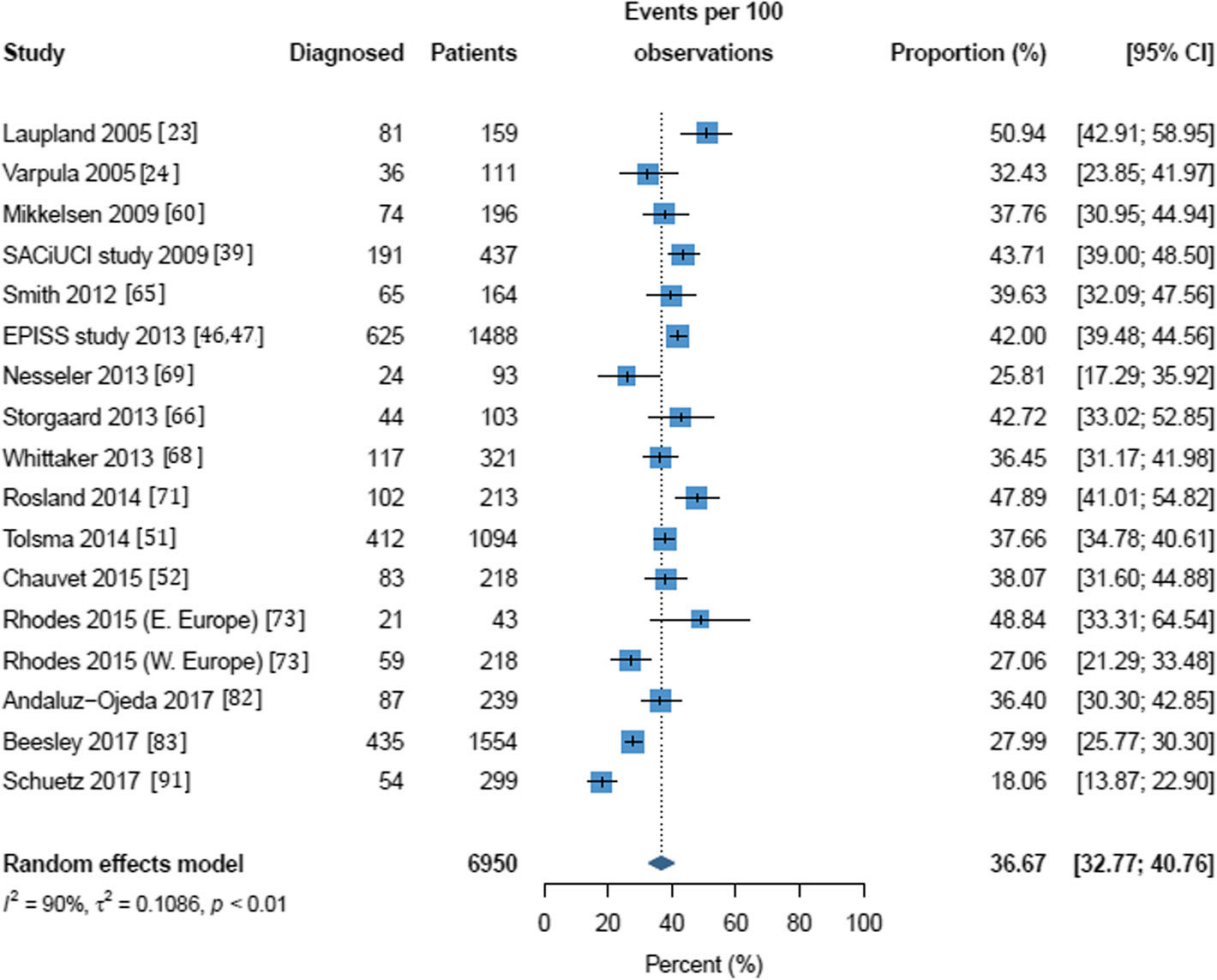
Random effects meta-analysis of studies reporting 28-/30-day mortality in septic shock patients. The forest plot contains exact 95% confidence intervals, and specific studies are weighted using the inverse-variance method. The pooled summary is obtained using the logit transformation and the DerSimonian–Laird method estimates between-study variance

Mortality rates in the included studies are reported in Additional file 1: Table S2. Three random-effects meta-analyses were performed to evaluate the mortality of septic shock in the ICU, in the hospital and at 28/30 days; these are shown in Figs. 3, 4, and 5. For seven of the publications reporting mortality, the number of patients could not be calculated and these were excluded from the meta-analyses [37, 39, 58, 67, 79, 88, 90]. One study reported a 1-day point prevalence estimate for hospital mortality and was excluded from the meta-analysis [34]. The mean mortality was 37.3% (95% CI 31.5% to 43.5%) in the ICU and 39.0% (95% CI 34.4% to 43.9%) in-hospital. Mortality at 28/30 days was estimated at 36.7% (95% CI 32.8% to 40.8%). Statistically significant heterogeneity was observed in all meta-analyses with reported *I*^2^ values of 98, 100, and 90 for in-ICU, in-hospital, and 28-/30-day mortality, respectively. ICU and hospital mortality estimates increased to 51.9% (95% CI 43.9 to 59.8%) and 52.1% (95% CI 51.6 to 52.6%) respectively when septic shock was diagnosed using Sepsis-3 criteria. As with frequency, estimates for mortality in the ICU and hospital and at 28/30 days were all higher in European populations than in North American populations (37.9% vs. 32.8%, 42.7% vs. 32.3%, and 38.5% vs. 33.2%) though not significantly so (see Additional file 1: Figures S6–S8). Estimates for ICU, hospital, and 28-/30-day mortality were higher in multicenter than in single-center studies (41.8% vs 37.1%, 43.1% vs. 34.0%, and 37.2% vs. 36.2%, respectively; Additional file 1: Figures S9-S11); none of these differences were statistically significant.

## Discussion

Our systematic review shows that approximately 10% of ICU patients have septic shock at admission and 8% of patients admitted to the ICU have septic shock sometime during their ICU stay. Populations assessed using Sepsis-3 criteria had a lower estimate at admission (approximately 7%). This may be a result of the narrower definition used in comparison to previous consensus criteria [92]. Earlier versions of the sepsis consensus definitions did not take into account lactate levels and required that patients met 2 or more systemic inflammatory response syndrome (SIRS) criteria; therefore, they are likely to be more inclusive [38, 93, 94]. The high occurrence of septic shock among ICU patients supports current efforts to raise awareness of the condition among healthcare professionals and the general public, but caution should be exercised when interpreting our pooled estimates given the high heterogeneity observed between studies. This variability in estimates could be driven by patient populations and, as the European- and North American-specific estimates suggest, country of origin. The different methods of identifying cases, using discharge codes, administrative data, or electronic chart reviews, might also account for some of the differences. For example, some coding abstraction methods may underestimate the diagnosis [95] and significant numbers of patients may be misclassified by electronic health record-based definitions when the frequency of the condition is low [96]. Additionally, the higher number of septic shock cases reported in some countries may partly be explained by the lower number of ICU beds resulting in a concentration of more severely ill patients. For example, a study of Portuguese ICUs revealed an unusually high frequency of septic shock diagnosed at admission (approximately 23%) that the authors suggested was most probably related to the lack of ICU services in Portugal [50]. The differences observed might also be due to seasonal variations in the epidemiology of septic shock as the time period of the studies varied widely from a single day to several years. Additionally, the reporting of the data was not always satisfactory. Many studies collected data from a mix of patients diagnosed either at admission or screened prospectively during their ICU stay. These data were often not reported separately, so our estimates may be a mix of frequency and incidence rates.

Our results show that, regardless of the time-point of assessment, the septic shock mortality rate is approximately 38%. This is somewhat lower than the results of the systematic review conducted by Shankar-Hari et al. that estimated a septic shock-associated crude mortality of 46.5% [15]. This may be a result of the inclusion of some long-term follow-up data by Shankar-Hari et al. whereas the current review focused on short-term mortality. Moreover, the Shankar-Hari review included studies in which patient data were collected prior to 1995 when mortality rates may have been higher on average than in more recent studies. Indeed, 20–25 years ago, non-selected populations had a crude mortality rate for septic shock of more than 50% [97, 98]. In the years since, observational studies have reported higher survival rates [7, 46, 99–102], likely because of improvements in the general management of patients with septic shock. Our estimates for 28–30-day mortality are higher than those identified by a systematic review on long-term mortality [103], which reported post-acute phase mortality (difference in proportion between cumulative 1-year mortality and acute mortality) of 16.1% (95% CI 14.1 to 18.1%), levels that are likely to incur a high burden on overall costs and quality of life. Estimates for ICU and in-hospital mortality were higher using Sepsis-3 criteria than non-Sepsis-3 criteria (52% vs. 37% and 52% vs. 39%), which may be a result of the greater severity of disease associated with the addition of hyperlactatemia as a component for septic shock diagnosis. This is consistent with previous research that found that Sepsis-3 identified patients who were more advanced in the course of their disease and in whom a poor outcome was more likely than in those identified using Sepsis-2 criteria [104]. Our results thus support previous studies postulating that the significant shift in the diagnostic criteria from SIRS criteria to a focus on organ dysfunction would have a major impact on mortality [38, 94].

An up-to-date understanding of the incidence of septic shock and associated mortality is important to help guide resource allocation and inform healthcare budgets. Indeed, the economic costs of sepsis are a significant burden on healthcare systems. In the USA, approximately US$20 billion is spent annually on hospital care for sepsis patients, with septic shock requiring longer ICU care and higher hospitalization costs [105]. In the UK, recent data suggest total annual hospital costs of just over £1 billion for patients with sepsis [106]. The effects of sepsis on patient health last beyond hospital discharge, with an increased mortality risk for years after hospitalization [107–109]. Nearly a quarter of sepsis survivors are readmitted to hospital within 30 days of discharge [110], and survivors often exhibit profound immune suppression, physical and psychological disorders, and impaired quality of life [107, 109, 111]. These long-term consequences greatly contribute to the high total economic cost of the disease, which is estimated to be around US$67 billion yearly in the USA alone [105]. In the UK, the total annual costs of sepsis taking into account these indirect costs are estimated at around £10 billion [106].

The results of our review should be interpreted with consideration of certain limitations. Firstly, we only included studies published in English, potentially leading to language bias as relevant studies published in other languages were not included. Secondly, estimates using Sepsis-3 criteria were derived from very few studies.

## Conclusion

Our findings suggest that septic shock occurs frequently in ICU patients and mortality remains high. Considerable differences in diagnostic criteria have been employed in the different studies over the last 15 years. Our review indicates that the adoption of Sepsis-3 criteria is still at an early stage (≤ 3 studies); however, as Sepsis-3 is increasingly adopted and more epidemiological studies are published in which these criteria are used, a more complete picture will emerge concerning what differences, if any, exist between countries and different care settings. This would allow a more accurate estimation of the burden of septic shock, thus enabling the development of better policies for public health planning and hospital management.

## Additional file

Additional file 1: Search strategy and inclusion and exclusion criteria, **Tables S1–S4** and **Figures S1–S11.** (PDF 1970 kb)

## Abbreviations

CI: Confidence interval
EPISS: EPIdemiology of Septic Shock study
ICD: International Classification of Diseases
ICU: Intensive care unit
MeSH: Medical Subject Headings
PRISMA-P: Preferred Reporting Items for Systematic Review and Meta-Analysis Protocols
SACiUCI: Portuguese Community-Acquired Sepsis study
SIRS: Systemic inflammatory response syndrome
SOAP: Sepsis Occurrence in Acutely Ill Patients study
