# Supplementary material for: Frequency and mortality of septic shock in Europe and North America: a systematic review and meta-analysis
Source: Crit Care. 2019 May 31;23:196. doi: 10.1186/s13054-019-2478-6 (PMC6545004; doi:10.1186/s13054-019-2478-6)
Supplement: Supplementary file 1 — Search strategy and inclusion and exclusion criteria, Tables S1–S4 and Figures S1–S11. (PDF 1970 kb) [file 13054_2019_2478_MOESM1_ESM.pdf]

Frequency and mortality of septic shock in Europe and North America: a systematic review and meta-analysis

Jean-Louis Vincent et al.

Additional File 1

Search strategy

Medline and Medline In-Process

Searched 23/12/15 via OvidSP interface.
Limited to 2005 onwards, no language limits.
Database: Ovid MEDLINE(R) In-Process & Other Non-Indexed Citations and Ovid MEDLINE(R) <1946 to Present>
Search strategy:
1 septic shock/ (19631)
2 ((seps$ or sept$) adj3 (shock$ or severe or syndrome$)).ti,ab. (25875)
3 ((bacteremic or bacterial or bacteriemic or endotoxic or endotoxine or toxic) adj2 shock).ti,ab. (6661)
4 ("forward failure" adj2 toxic).ti,ab. (0)
5 1 or 2 or 3 or 4 (39398)
6 *Epidemiology/ (9719)
7 epidemiology.fs. (1336638)
8 6 or 7 (1345469)
9 quality-adjusted life years/ or quality of life/ (142229)
10 (sf36 or sf 36 or sf-36 or short form 36 or shortform 36 or sf thirtysix or sf thirty six or shortform thirtysix or shortform thirty six or short form thirty six or short form thirtysix or short form thirty six).ti,ab. (18820)
11 (sf6 or sf 6 or sf-6 or short form 6 or shortform 6 or sf six or sfsix or shortform six or short form six).ti,ab. (1540)
12 (sf12 or sf 12 or sf-12 or short form 12 or shortform 12 or sf twelve or sftwelve or shortform twelve or short form twelve).ti,ab. (3542)
13 (sf6D or sf 6D or sf-6D or short form 6D or shortform 6D or sf six D or sfsixD or shortform six D or short form six D).ti,ab. (562)
14 (sf20 or sf 20 or sf-20 or short form 20 or shortform 20 or sf twenty or sftwenty or shortform twenty or short form twenty).ti,ab. (359)
15 (sf8 or sf 8 or sf-8 or short form 8 or shortform 8 or sf eight or sfeight or shortform eight or short form eight).ti,ab. (330)
16 "health related quality of life".ti,ab. (27384)
17 (Quality adjusted life or Quality-adjusted-life).ti,ab. (7931)
18 "assessment of quality of life".ti,ab. (1343)
19 (euroqol or euro qol or eq5d or eq 5d).ti,ab. (5479)
20 (hql or hrql or hqol or h qol or hrqol or hr qol).ti,ab. (12836)
21 (hye or hyes).ti,ab. (63)
22 health$ year$ equivalent$.ti,ab. (40)
23 (hui or hui1 or hui2 or hui3 or hui4 or hui-4 or hui-1 or hui-2 or hui-3).ti,ab. (1066)
24 (quality time or qwb or quality of well being or "quality of wellbeing" or "index of wellbeing" or "index of well being").ti,ab. (701)
25 (Disability adjusted life or Disability-adjusted life or health adjusted life or health-adjusted life or "years of healthy life" or healthy years equivalent or "years of potential life lost" or "years of health life lost").ti,ab. (2348)
26 (QALY$ or DALY$ or HALY$ or YHL or HYES or YPLL or YHLL or qald$ or qale$ or qtime$ or AQoL$).ti,ab. (8863)

27 (timetradeoff or time tradeoff or time trade-off or time trade off or TTO or Standard gamble$ or "willingness to pay").ti,ab. (4726)
28 15d.ti,ab. (1355)
29 (HSUV$ or health state$ value$ or health state$ preference$ or HSPV$).ti,ab. (283)
30 (utilit$ adj3 ("quality of life" or valu$ or scor$ or measur$ or health or life or estimat$ or elicit$ or disease$)).ti,ab. (8259)
31 (utilities or disutili$).ti,ab. (5010)
32 (Severity Weighted Assessment Tool or SWAT or mSWAT).ti,ab. (402)
33 exp Health Behavior/ (132133)
34 (patient$ adj2 (attitude$ or compliance or "non compliance" or adheren$ or "non adherence" or participation or "non participation" or preference$ or satisf$ or dissatisf$ or toleran$ or intoleran$ or "reported outcome" or "reported outcomes")).ab,ti. (80522)
35 or/9-34 (367715)
36 economics/ (27237)
37 exp "costs and cost analysis"/ (196980)
38 economics, dental/ (1889)
39 exp "economics, hospital"/ (21044)
40 economics, medical/ (9044)
41 economics, nursing/ (3971)
42 economics, pharmaceutical/ (2657)
43 (economic$ or cost or costs or costly or costing or price or prices or pricing or pharmacoeconomic$).ti,ab. (541242)
44 (expenditure$ not energy).ti,ab. (21208)
45 (value adj1 money).ti,ab. (28)
46 budget$.ti,ab. (21496)
47 36 or 37 or 38 or 39 or 40 or 41 or 42 or 43 or 44 or 45 or 46 (676152)
48 ((energy or oxygen) adj cost).ti,ab. (3195)
49 (metabolic adj cost).ti,ab. (966)
50 ((energy or oxygen) adj expenditure).ti,ab. (19253)
51 48 or 49 or 50 (22612)
52 47 not 51 (671059)
53 exp Employment/ (59564)
54 exp Work/ (15209)
55 Efficiency/ (11965)
56 Absenteeism/ (7756)
57 "Cost of Illness"/ (20490)
58 "Length of Stay"/ (66244)
59 ((employment or employed or employee$ or unemployment or unemployed) adj3 (economic$ or cost or costs or costly or costing or price or prices or pricing)).ti,ab. (1690)
60 (productivity adj3 (economic$ or cost or costs or costly or costing or price or prices or pricing)).ti,ab. (2051)
61 ((long standing or longstanding or long term or longterm or permanent or employee$) adj2 (absence$ or absent$ or ill$ or sick$ or disab$)).ti,ab. (7971)
62 llsi.ti,ab. (11)
63 (cost$ adj2 (illness or disease$ or sickness$)).ti,ab. (3558)
64 (burden$ adj2 (disease$ or illness or sickness$)).ti,ab. (14924)
65 ((social or societ$ or work$ or employe$ or business$ or communit$ or famil$ or carer$ or caregiver$) adj3 (burden$ or consequenc$ or impact$ or problem$ or productivity or sickness or impairment$)).ti,ab. (64832)
66 ((allowance or status or long-term or pension$ or benefit$) adj2 disab$).ti,ab. (8851)
67 ((unable or inability or incapacit$ or incapab$) adj3 work).ti,ab. (1492)
68 budget$ impact$.ti,ab. (732)
69 budget$ implicat$.ti,ab. (44)
70 resource$ use$.ti,ab. (6852)
71 resource$ utili$.ti,ab. (6485)

72 resource$ usage.ti,ab. (239)
73 (length adj2 stay$).ti,ab. (34475)
74 (hospital$ adj2 stay$).ti,ab. (62003)
75 (duration adj2 stay$).ti,ab. (2462)
76 extended stay$.ti,ab. (137)
77 prolonged stay$.ti,ab. (699)
78 ((hospitali?ation or hospitali?ed) adj3 (economic$ or cost or costs or costly or costing or price or prices or pricing)).ti,ab. (4251)
79 economic consequenc$.ti,ab. (2609)
80 or/52-79 (916218)
81 Critical Pathways/ (5192)
82 practice guideline/ (21297)
83 (guideline$ or guidance or ((care or clinical or critical) adj1 pathway$)).ti,ab. (297939)
84 or/81-83 (310152)
85 5 and 8 (3130)
86 5 and 35 (208)
87 5 and 80 (1783)
88 5 and 84 (913)
89 85 or 86 or 87 or 88 (5247)
90 89 not (animals/ not (human/ and animals/)) (5175)
91 90 not (editorial or letter or case reports).pt. (4747)
92 limit 91 to yr="2005 -Current" (3163)
3163 results.

Embase
Searched 23/12/15 via OvidSP interface.
Limited to 2005 onwards, no language limits.
Database: Embase <1996 to 2015 Week 51>
Search strategy:
1 septic shock/ (28007)
2 ((seps$ or sept$) adj3 (shock$ or severe or syndrome$)).ti,ab. (31144)
3 ((bacteremic or bacterial or bacteriemic or endotoxic or endotoxine or toxic) adj2 shock).ti,ab. (4448)
4 ("forward failure" adj2 toxic).ti,ab. (0)
5 or/1-4 (44748)
6 *epidemiology/ (23866)
7 *epidemiological data/ (1479)
8 ep.fs. (686321)
9 or/6-8 (709649)
10 quality adjusted life year/ or quality of life index/ (17105)
11 Short Form 12/ or Short Form 20/ or Short Form 36/ or Short Form 8/ (17502)
12 "International Classification of Functioning, Disability and Health"/ or "Ferrans and Powers Quality of Life Index"/ (1677)
13 (sf36 or sf 36 or sf-36 or short form 36 or shortform 36 or sf thirtysix or sf thirty six or shortform thirtysix or shortform thirty six or short form thirty six or short form thirtysix or short form thirty six).ti,ab. (27627)
14 (sf6 or sf 6 or sf-6 or short form 6 or shortform 6 or sf six or sfsix or shortform six or short form six).ti,ab. (1163)
15 (sf12 or sf 12 or sf-12 or short form 12 or shortform 12 or sf twelve or sftwelve or shortform twelve or short form twelve).ti,ab. (5424)
16 (sf6D or sf 6D or sf-6D or short form 6D or shortform 6D or sf six D or sfsixD or shortform six D or short form six D).ti,ab. (914)
17 (sf20 or sf 20 or sf-20 or short form 20 or shortform 20 or sf twenty or sftwenty or shortform twenty or short form twenty).ti,ab. (295)

18 (sf8 or sf 8 or sf-8 or short form 8 or shortform 8 or sf eight or sfeight or shortform eight or short form eight).ti,ab. (511)
19 "health related quality of life".ti,ab. (36206)
20 (Quality adjusted life or Quality-adjusted-life).ti,ab. (10847)
21 "assessment of quality of life".ti,ab. (1869)
22 (euroqol or euro qol or eq5d$ or eq 5d$).ti,ab. (9628)
23 (hql or hrql or hqol or h qol or hrqol or hr qol).ti,ab. (19034)
24 (hye or hyes).ti,ab. (73)
25 health$ year$ equivalent$.ti,ab. (25)
26 (hui or hui1 or hui2 or hui3 or hui4 or hui-4 or hui-1 or hui-2 or hui-3).ti,ab. (1398)
27 (quality time or qwb or "quality of well being" or "quality of wellbeing" or "index of wellbeing" or index of well being).ti,ab. (737)
28 (Disability adjusted life or Disability-adjusted life or health adjusted life or health-adjusted life or "years of healthy life" or healthy years equivalent or "years of potential life lost" or "years of health life lost").ti,ab. (2460)
29 (QALY$ or DALY$ or HALY$ or YHL or HYES or YPLL or YHLL or qald$ or qale$ or qtime$ or AQoL$).ti,ab. (13752)
30 (timetradeoff or time tradeoff or time trade-off or time trade off or TTO or Standard gamble$ or "willingness to pay").ti,ab. (6463)
31 15d.ti,ab. (1821)
32 (HSUV$ or health state$ value$ or health state$ preference$ or HSPV$).ti,ab. (354)
33 (utilit$ adj3 ("quality of life" or valu$ or scor$ or measur$ or health or life or estimat$ or elicit$ or disease$)).ti,ab. (11256)
34 (utilities or disutili$).ti,ab. (6843)
35 (Severity Weighted Assessment Tool or SWAT or mSWAT).ti,ab. (507)
36 "patient attitude"/exp or (patient$ adj2 (attitude$ or compliance or "non compliance" or adheren$ or "non adherence" or participation or "non participation" or preference$ or satisf$ or dissatisf$ or toleran$ or intoleran$ or "reported outcome" or "reported outcomes")).ab,ti. (102459)
37 or/10-36 (206261)
38 health economics/ (17272)
39 exp economic evaluation/ (202530)
40 exp "health care cost"/ (196101)
41 exp pharmacoeconomics/ (141614)
42 (econom$ or cost or costs or costly or costing or price or prices or pricing or pharmacoeconomic$).ti,ab. (576790)
43 (expenditure$ not energy).ti,ab. (21702)
44 (value adj2 money).ti,ab. (1435)
45 budget$.ti,ab. (20666)
46 or/38-45 (817477)
47 (metabolic adj cost).ti,ab. (815)
48 ((energy or oxygen) adj cost).ti,ab. (2250)
49 ((energy or oxygen) adj expenditure).ti,ab. (18776)
50 or/47-49 (21261)
51 46 not 50 (813441)
52 exp employment/ (40916)
53 exp work/ (197303)
54 "cost of illness"/ (15399)
55 "length of stay"/ (99592)
56 ((employment or employed or employee$ or unemployment or unemployed) adj3 (economic$ or cost or costs or costly or costing or price or prices or pricing)).ti,ab. (1614)
57 (productivity adj3 (economic$ or cost or costs or costly or costing or price or prices or pricing)).ti,ab. (2401)
58 ((long standing or longstanding or long term or longterm or permanent or employee$) adj2 (absence$ or absent$ or ill$ or sick$ or disab$)).ti,ab. (8459)
59 llsi.ti,ab. (11)

60 (cost$ adj2 (illness or disease$ or sickness$)).ti,ab. (4623)
61 (burden$ adj2 (disease$ or illness or sickness$)).ti,ab. (20518)
62 ((social or societ$ or work$ or employe$ or business$ or communit$ or famil$ or carer$ or caregiver$) adj3 (burden$ or consequenc$ or impact$ or problem$ or productivity or sickness or impairment$)).ti,ab. (69824)
63 ((social or societ$ or work$ or employe$ or business$ or communit$ or famil$ or carer$ or caregiver$) adj3 (burden$ or consequenc$ or impact$ or problem$ or productivity or sickness or impairment$)).ti,ab. (69824)
64 ((allowance or status or long-term or pension$ or benefit$) adj2 disab$).ti,ab. (11494)
65 ((unable or inability or incapacit$ or incapab$) adj3 work).ti,ab. (1370)
66 budget$ impact$.ti,ab. (1920)
67 budget$ implicat$.ti,ab. (53)
68 resource$ use$.ti,ab. (9170)
69 resource$ utili$.ti,ab. (10271)
70 resource$ usage.ti,ab. (317)
71 (length adj2 stay$).ti,ab. (54025)
72 (hospital$ adj2 stay$).ti,ab. (84781)
73 (duration adj2 stay$).ti,ab. (3112)
74 extended stay$.ti,ab. (169)
75 prolonged stay$.ti,ab. (829)
76 ((hospitali?ation or hospitali?ed) adj3 (economic$ or cost or costs or costly or costing or price or prices or pricing)).ti,ab. (6094)
77 economic consequenc$.ti,ab. (2865)
78 or/51-77 (1212529)
79 clinical pathway/ or practice guideline/ or (guideline$ or guidance or ((care or clinical) adj1 pathway$)).ti,ab. (527797)
80 5 and 9 (2278)
81 5 and 37 (278)
82 5 and 78 (4210)
83 5 and 79 (2423)
84 or/80-83 (7991)
85 84 not (animals/ not (human/ and animals/)) (7976)
86 85 not ((editorial or letter or conference or "conference paper" or "conference proceeding" or "conference proceeding article" or "conference proceeding conference paper" or "conference proceeding editorial" or "conference proceeding note" or "conference proceeding review" or "journal conference abstract" or "journal conference paper" or "journal conference review").pt. or case report/) (5073)
87 limit 86 to yr="2005 -Current" (4013)
88 limit 87 to embase (3428)
3428 results.

Cochrane Library
Includes Cochrane Database of Systematic Reviews (CDSR), Database of Abstracts of Reviews of Effects (DARE), Cochrane Central Register of Controlled Trials (CENTRAL), Health Technology Assessment Database (HTAD), NHS Economic Evaluations Database (NHSEED).
Searched 23/12/15 online at http://onlinelibrary.wiley.com/cochranelibrary/search/advanced
Search strategy:
#1 MeSH descriptor: [Shock, Septic] this term only (451)
#2 ((seps* or sept* or septicem* or septicaem*) near/3 (shock* or severe or syndrome*)):ti,ab (1570)
#3 ((bacteremic or bacterial or bacteriemic or endotoxic or endotoxine or toxic) near/2 shock):ti,ab (24)
#4 ("forward failure" near/2 toxic):ti,ab (0)
#5 #1 or #2 or #3 or #4 Publication Year from 2005 to 2015 (1013)

1013 total results comprised of 27 from CDSR, 51 from DARE, 895 from CENTRAL, 10 from HTAD, and 25 from NHSEED.

Update of epidemiology searches February 2018

The epidemiology sections of the original searches were re-run from the date of the original searches onwards;

Medline and Medline In-Process

Searched 20/02/18 via OvidSP interface.
Limited to 2005 onwards, no language limits.
Limited to records added to database from 23/12/15 (date of previous search) onwards.
Database: Ovid MEDLINE(R) Epub Ahead of Print, In-Process & Other Non-Indexed Citations, Ovid MEDLINE(R) Daily and Ovid MEDLINE(R) <1946 to Present>
Search Strategy:
1 septic shock/ (20578)
2 ((seps$ or sept$) adj3 (shock$ or severe or syndrome$)).ti,ab. (29380)
3 ((bacteremic or bacterial or bacteriemic or endotoxic or endotoxine or toxic) adj2 shock).ti,ab. (6935)
4 ("forward failure" adj2 toxic).ti,ab. (0)
5 1 or 2 or 3 or 4 (43239)
6 *Epidemiology/ (9582)
7 epidemiology.fs. (1455855)
8 6 or 7 (1464532)
9 5 and 8 (3558)
10 9 not (animals/ not (human/ and animals/)) (3526)
11 10 not (editorial or letter or case reports).pt. (3285)
12 limit 11 to yr="2005 -Current" (2207)
13 ("20151223" or "20151224" or "20151225" or "20151226" or "20151228" or "20151229" or "20151230" or "20151231" or 2016$ or 2017$ or 2018$).ed. (2093879)
14 12 and 13 (526)

Embase

Searched 20/02/18 via OvidSP interface.
Limited to 2005 onwards, no language limits.
Limited to records added to database from 23/12/15 (date of previous search) onwards.
Database: Embase <1996 to 2018 Week 08>
Search Strategy:
1 septic shock/ (35404)
2 ((seps$ or sept$) adj3 (shock$ or severe or syndrome$)).ti,ab. (38557)
3 ((bacteremic or bacterial or bacteriemic or endotoxic or endotoxine or toxic) adj2 shock).ti,ab. (4914)
4 ("forward failure" adj2 toxic).ti,ab. (0)
5 or/1-4 (55133)
6 *epidemiology/ (28307)
7 *epidemiological data/ (1867)
8 ep.fs. (742580)
9 or/6-8 (770558)
10 5 and 9 (2544)
11 10 not (animals/ not (human/ and animals/)) (2535)
12 11 not ((editorial or letter or conference or "conference paper" or "conference proceeding" or "conference proceeding article" or "conference proceeding conference paper" or "conference proceeding editorial" or "conference proceeding note" or "conference proceeding review" or "journal conference abstract" or "journal conference paper" or "journal conference review").pt. or case report/) (2163)

13 limit 12 to yr="2005 -Current" (1578)
14 limit 13 to embase (1284)
15 ("20151223" or "20151224" or "20151225" or "20151226" or "20151227" or "20151228" or "20151229" or "20151230" or "20151231" or 2016$ or 2017$ or 2018$).dc. (3552842)
16 14 and 15 (240)

Cochrane Library

Includes Cochrane Database of Systematic Reviews (CDSR), Database of Abstracts of Reviews of Effects (DARE), Health Technology Assessment Database (HTAD), NHS Economic Evaluations Database (NHSEED). CENTRAL was not included for this epidemiology update- only reviews, HTA reports, or economic evaluations that might contain epidemiology sections.
Searched 21/02/18 online at http://onlinelibrary.wiley.com/cochranelibrary/search/advanced
Search strategy:
#1 MeSH descriptor: [Shock, Septic] this term only (565)
#2 ((seps* or sept* or septicem* or septicaem*) near/3 (shock* or severe or syndrome*)):ti,ab (2294)
#3 ((bacteremic or bacterial or bacteriemic or endotoxic or endotoxine or toxic) near/2 shock):ti,ab (32)
#4 ("forward failure" near/2 toxic):ti,ab (0)
#5 #1 or #2 or #3 or #4 Publication Year from 2016 to 2018, in Cochrane Reviews (Reviews and Protocols), Other Reviews, Technology Assessments and Economic Evaluations (9)
9 total results comprised of 9 from CDSR.

Inclusion and exclusion criteria for systematic review of epidemiology of septic shock

Inclusion criteria

(1) Observational studies of frequency of septic shock in an assessed cohort ≥100 patients
(2) Observational studies reporting the mortality of septic shock in an assessed cohort ≥15 patients
(3) Studies published in the last 13 years (2005 – 2018)
(4) Studies reporting information for one of the following countries: USA, Canada and any European country

Exclusion criteria

(1) Pediatric patients (aged <15 years) with sepsis/septic shock
(2) Non-observational/interventional studies (RCTs, quasi-RCT)
(3) Studies with frequency /incidence/mortality of septic shock in specific disease groups/special populations (e.g. cancer, obesity, HIV, pregnancy).
(4) Studies in which the aim is to assess the effect of pharmacological interventions
(5) Studies indexed as case reports, editorials and letters
(6) Conference abstracts
(7) Non-human studies
(8) Non-English language publications
(9) Studies published before 2005
(10) Studies reporting information for countries other than USA, Canada and Europe

Figure S1. Preferred Reporting Items for Systematic Reviews and Meta-Analyses (PRISMA) flow chart for the systematic literature review of septic shock

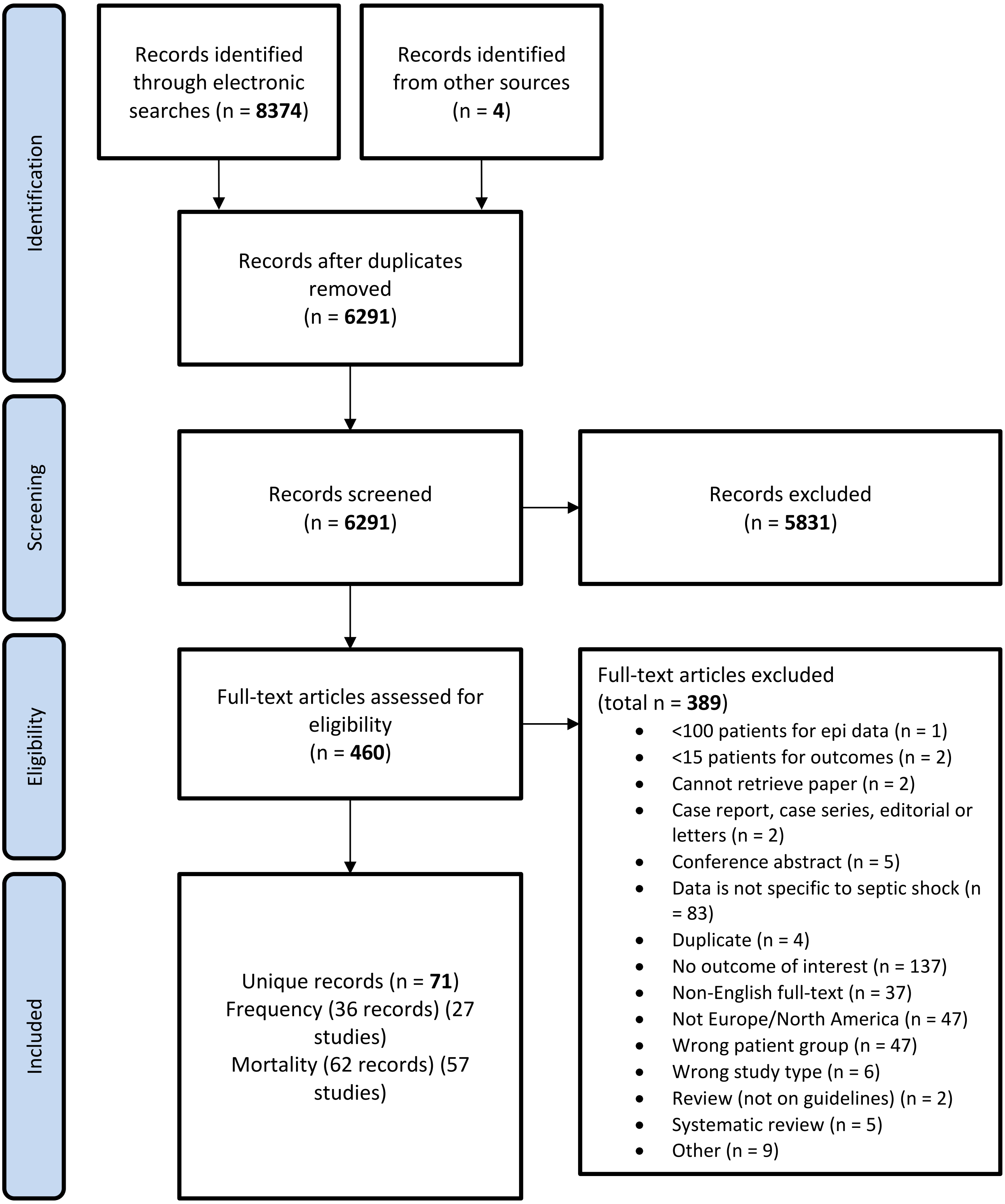

Table S1. Summary of included studies reporting septic shock frequency

	Author, Year [ref]	Time period	No. of centers	Cohort screened	Timepoint for septic shock diagnosis	Frequency: cases/total number screened (%)	Criteria used for diagnosis	Data source
France	Adrie 2005 [22]	1997 – 2000	6	All adult admissions hospitalized for more than 48 hours	Anytime during stay	279/1,698 (16.4)	Not reported	OUTCOMEREA database generated from standardized report forms
Canada	Laupland 2005 [23]	1999 – 2002	7	All adult admissions	At admission	159/4,845 (3.3)	BSI-associated sepsis requiring vasopressor infusion	Four large regional databases
Finland	Varpula 2005 [24]	1999 – 2002	1	Consecutive admissions	Anytime during stay	111/1,419 (7.8)	Discharge diagnosis compatible with sepsis and a need for vasopressor support during the first 48 h	ICU clinical data management system (HP CareVue, Palo Alto, Calif., USA)
Croatia	Degoricija 2006 [25]	2000 – 2005	1	Medical patients referred from the emergency department	At admission	125/5,022 (2.5)	SEPSIS 1/2	Hospital database
					Anytime during stay	138/5,022 (2.7)		
Multiple European	SOAP study (Sakr 2006 [27], Vincent 2006 [26], Sprung 2006 [28], Sakr 2007 [32])	2002	198	Consecutive adult admissions	At admission	243/3,147 (7.7)	SEPSIS 1	Case report forms entered in a central database
					Anytime during stay	462/3,147 (14.7)		
France	Guidet 2007 [30]	1998 – 2002	35	Admissions in the CUB-Rea network	Anytime during stay	4,707/98,015 (4.8)	Not reported	Cub-REA database
Germany	Engel 2007 [29], Elke 2008 [34]	2003 – 2004	454	All patients present on a single day in 310 randomly chosen hospitals	Anytime during stay	Engel: 190/3,877 (4.9)<br>Elke: 184/3,877 (4.7)	SEPSIS 1 (modified)	Case report forms entered into a national central database
Finland	Karlsson 2007 [31]	2004 – 2005	25	Consecutive adult admissions	Anytime during stay	363/4,500 (8.1)	SEPSIS 1	Hospital admissions and discharge data
Spain	Blanco 2008 [33]	2002	14	Consecutive admissions (without non-infectious heart problems)	Anytime during stay	180/2,619 (6.9)	Not reported	Standardized forms entered into a multicenter central database
Italy	Malacarne 2008 [35]	2002 – 2003	71	Consecutive admissions (>48 hrs stay)	Anytime during stay	224/5,814 (3.9)	SEPSIS 2	Electronic case record forms entered into a multicenter national database
France	Adrie 2009 [36]	1996 – 2007	12	Consecutive adult admissions hospitalized	Anytime during stay	674/7,719 (8.7)	SEPSIS 1	OUTCOMEREA database generated from

				for more than 24 hours				standardized report forms
Germany	Weiss 2009 [38]	2007	1	Surgical critically ill patients admitted to an adult ICU	Anytime during stay	162/742 (21.8)	SEPSIS 1	Standardized electronic case report forms entered into a single center database
						205/742 (27.6)	SEPSIS 2	Standardized electronic case report forms entered into a single center database
Portugal	SACiUCI study (Povoa 2009 [37], Cardoso 2010 [39])	2004 – 2005	17	Consecutive adult admissions	At admission	Povoa: 458/4,142 (11.1)<br><br>Cardoso: 437/4,142 (10.6)	SEPSIS 1/2 (Povoa)<br>SEPSIS 1 (Cardoso)	Data collected prospectively into a multicenter national database
Austria	Wurzinger 2010 [40]	2003 – 2008	1	Prospectively collected database of admissions	Anytime during stay	343/2,700 (12.7)	SEPSIS 2	Institutional database and medical records
USA	Moore 2011 [41]	2007 – 2009	1	Retrospective screen of all admissions	Anytime during stay	61/4,514 (1.4)	SEPSIS 1 (modified)	General surgical ICU database
USA	Plataki 2011 [42]	2005 – 2007	1	Consecutive adult (>18 years of age) patients admitted to a medical ICU	Anytime during stay	467/4,893 (9.4)	SEPSIS 1	Retrospective review of medical records
Netherlands	Klein Klouwenberg 2012 [43]	2009 – 2010	1	All adult admissions (not transferred or elective surgery)	At admission	98/1,072 (9.1)	Liberal definition: sepsis (≥2 criteria transiently present during a 24 h period of automatic recording) plus refractory hypotension	Clinical data collected prospectively
							Restrictive definition: sepsis (≥3 criteria simultaneously present with manual recording at hourly intervals) plus refractory hypotension with other organ system failure	
Portugal	Almeida 2013 [44]	2004 – 2008	1	Consecutive adult admissions	Anytime during stay	197/1,223 (16.1)	SEPSIS 1	Clinical data registered prospectively until hospital discharge
France	EPISS study (Pavon 2013 [46], Quenot 2013 [47])	2009 – 2011	14	Consecutive adult admissions	Anytime during stay	1,495/10,941 (13.7)	Infection requiring vasopressors despite adequate vascular filling with either metabolic acidosis, oliguria/renal insufficiency or hepatic	Clinical data registered prospectively using standardized electronic case report forms

							dysfunction	
USA	Miller 2013 [45]	2004 – 2010	18	Consecutive admissions	At admission	1,426/15,019 (9.5)	SEPSIS 2	Retrospective chart review and electronic health records
Italy	PICU-Network study (Sakr 2013 [48], Sakr 2013 [49])	2006	24	All adult patients admitted to a network of 24 ICUs from 17 hospitals during a 180 day period	At admission	100/3,902 (2.6)	SEPSIS 1	Electronic case record forms entered into a multicenter regional database
					Anytime during stay	145/3,902 (3.7)		
Portugal	INFAUCI study Goncalves-Pereira 2014 [50]	2009 – 2010	14	Consecutive adult admissions	At admission	856/3,766 (22.7)	SEPSIS 1/2	Data collected prospectively into a multicenter database
France	Tolsma 2014 [51]	1997 – 2011	11	All patients admitted to ICU OUTCOMEREA database	At admission	1,094/14,419 (7.6)	Not reported	OUTCOMEREA database generated from standardized report forms
France	Chauvet 2015 [52]	2008 – 2010	1	Consecutive admissions	At admission	218/962 (22.7)	Not reported	Medical records and ICU observation charts
Poland	Kubler 2015 [53]	2012, 2013	NR	All patients treated on a single day of questionnaire completion	Anytime during stay	243/2,258 (10.8)	Surviving Sepsis Campaign 2008	National survey of ICUs
Greece	Papadimitriou-Olivgeris 2016 [55]	2011 – 2014	1	All adult admissions	At admission	55/834 (6.6)	SEPSIS 3	Retrospective chart review of a single center database
					Anytime during stay	136/834 (16.3)		
Germany	SepNet 2016 [54]	2013	154	All admissions	At admission	SEPSIS 1: 1,285/11,883 (10.8)<br><br>SEPSIS 3: 848/11,883 (7.1)	SEPSIS 1&3	Data collected prospectively
Netherlands	van Vught 2016 [56]	2011 – 2013	2	All consecutive admissions (>48 hrs stay)	At admission	549/3,168 (17.3)	Shock was defined by the use of noradrenaline for hypotension in a dose of more than 0.1 µg/kg/min during at least 50% of the ICU day	Data collected prospectively
UK	Shankar-Hari 2017 [57]	2011 – 2015	189	Consecutive adult admissions	At admission	SEPSIS 2: 153,257/654,918	SEPSIS 2&3	Data collected prospectively into a

						(23.4)<br><br>SEPSIS 3:<br>39,262/654,918<br>(6.0)		multicenter national database

ACCP/SCCM, American College of Chest Physicians/Society of Critical Care Medicine; BSI, bloodstream infection; ED, emergency department; EPISS; EPIdemiology of Septic Shock study; ICD, International classification of Diseases; ICU, intensive care unit; INFAUCI, The Infection on Admission to the ICU study; NR, not reported; PICU-Network, Piedmont Intensive Care Unit Network study; SACiUCI, Portuguese Community-Acquired Sepsis study; SIRS, systemic inflammatory response syndrome; SOAP, Sepsis Occurrence in Acutely Ill Patients study

Table S2. Summary of included studies reporting septic shock mortality outcomes

Country	Author, year [ref]	Timeframe of data collection	Setting (no. of centers)	Type of ICU	Criteria used	Data source	Cohort screened	Timepoint for septic shock diagnosis	Mortality Deaths/cases, n (%)
Canada	Laupland 2005 [23]	1999 – 2002	ICU (7)	Multi disciplinary, cardiovascular	BSI-associated sepsis requiring vasopressor infusion	Four large regional databases	All adult ICU admissions	At admission	28-day: 81/159 (50.9)
Finland	Varpula 2005 [24]	1999 – 2002	ICU (1)	Medical-surgical	Discharge diagnosis compatible with sepsis and a need for vasopressor support during the first 48 h	ICU clinical data management system (HP CareVue, Palo Alto, Calif., USA)	Consecutive ICU admissions	Anytime during stay	ICU: 33/111 (29.7)
									30-day: 36/111 (32.4)
									48-hr: 12/111 (10.8)
Croatia	Degoricija 2006 [25]	2000 – 2005	ICU (1)	Medical	SEPSIS 1/2	Hospital database	Medical ICU patients referred to the ICU from the emergency department	Anytime during stay	ICU: 107/138 (77.5)
Canada and USA	Kumar 2006 [58]	Cohort 1: 1999 – 2004<br>Cohort 2: 1989 – 1999<br>Cohort 3: 1999 – 2004	ICU (14); hospitals (10)	Medical, general surgical, mixed	SEPSIS 1	Local database where cases were encoded prospectively based on ICU registries and ICD-9 diagnoses	Hospital cohort data in locally developed databases (two Canadian cohorts and one American)	Anytime during stay	Hospital: NR/2731 (56.2)
Europe (multiple); SOAP study	Sakr 2006 [27], Vincent 2006 [26], Sprung 2006 [28], Sakr 2007 [32]	2002	ICU (198)	Not reported	SEPSIS 1	Case report forms entered in a central database	Consecutive adult ICU admissions	At admission	ICU: 111/243 (45.7)
									Hospital: 133/243 (54.7)

								Anytime during stay	ICU: 219/462 (47.4)
									Hospital: 250/462 (54.1)
Spain	Esteban 2007 [59]	2003	Hospital (3)	Medical-surgical	SEPSIS 1	Preprinted case report forms prospectively entered in a database until hospital discharge	Consecutive hospital admissions (> 48 hrs stay, not transferred with sepsis)	Anytime during stay	Hospital: 27/59 (45.8)
									ICU: 19/44 (43.2)
Finland	Karlsson 2007 [31]	2004 – 2005	ICU (25)	Multidisciplinary	SEPSIS 1	Hospital admissions and discharge data	Consecutive adult ICU admissions	Anytime during stay	ICU: 19/44 (43.2)
Germany	Elke 2008 [34]	2003	ICU (454)	Not reported	SEPSIS 1 (modified)	Case report forms entered into a national central database	All ICU patients present on a single day in 310 randomly chosen hospitals	Anytime during stay	Hospital: 110/177 (62.1)
Italy	Malacarne 2008 [35]	2002 – 2003	ICU (71)	Medical-surgical	SEPSIS 2	Electronic case record forms entered into a multicenter national database	Consecutive ICU admissions (>48 hrs stay)	Anytime during stay	Hospital: 358/472 (75.8)
									ICU: 334/472 (70.8)
USA	Mikkelsen 2009 [60]	2005 – 2006	ED (1)	Not reported	Hypotension (systolic blood pressure <90 mmHg) despite adequate fluid resuscitation or the use of vasoactive agents	Clinical data collected retrospectively from electronic medical records	ED admissions with severe sepsis	At admission	28-day: 74/196 (37.8)
Germany	Weiss 2009 [38]	2007	ICU (1)	Surgical	SEPSIS 1	Standardized electronic case report forms entered into a single center database	Surgical critically ill patients admitted to an adult ICU	Anytime during stay	ICU: 43/162 (26.5)
					SEPSIS 2	Standardized electronic case report forms entered into a single center database	Surgical critically ill patients admitted to an adult ICU	Anytime during stay	ICU: 45/205 (22.0)
Portugal	SACiUCI study (Povoa 2009 [37]; Cardoso 2010 [39])	2004 – 2005	ICU (17)	Medical, mixed	SEPSIS 1 (Cardoso), SEPSIS 1/2 (Povoa)	Data collected prospectively into a multicenter national database	Consecutive adult ICU admissions	At admission	ICU: NR/458 (44)
									28-day: 191/437 (43.7)
Spain	Pestana 2010 [61]	2003 – 2008	ICU (NR)	Surgical	SEPSIS 1	Review of medical records	Consecutive surgical ICU admissions with septic shock	Anytime during stay	ICU: 71/182 (39)

Austria	Wurzinger 2010 [40]	2003 – 2008	ICU (1)	Multi disciplinary	SEPSIS 2	Institutional database and medical records	Prospectively collected database of ICU admissions	Anytime during stay	ICU: 50/301 (16.6)<br>Hospital: 67/301 (22.3)
Spain	Castellanos-Ortega 2011 [63]	2005 – 2010	ICU (3)	Medical, surgical, transplant, neurology, neurosurgical, trauma	SEPSIS 2	Not reported	Consecutive adult admissions to medical-surgical ICUs with severe sepsis	At admission	Hospital: 165/546 (30.2)
Canada	Chua 2011 [64]	2006 – 2008	ICU (1)	Not reported	Not reported	Retrospective chart review	All ICU admissions	At admission	ICU: 52/150 (34.7)<br>Hospital: 66/150 (44.0)
USA	Moore 2011 [41]	2007 – 2009	ICU (1)	General surgery	SEPSIS 1 (modified)	A general surgical ICU database	Retrospective screen of all admissions	Anytime during stay	Hospital: 22/61 (36.1)<br>ICU: 17/61 (27.9)
USA	Plataki 2011 [42]	2005 – 2007	ICU (1)	Medical	SEPSIS 1	Retrospective review of medical records	Consecutive adult patients (>18 years of age) admitted to a medical ICU	Anytime during stay	Hospital: 167/390 (42.8)
France	Zahar 2011 [62]	1996 – 2009	ICU (12)	Not reported	Sepsis induced hypotension persisting despite adequate fluid resuscitation plus organ dysfunction	OUTCOMEREA database generated from standardized report forms	All patients admitted to ICU OUTCOMEREA database	Anytime during stay	Hospital (community): 215/530 (40.6)<br>Hospital (ICU): 123/232 (53.0)<br>Hospital (nosocomial): 233/480 (48.5)
Netherlands	Klein Klouwenberg 2012 [43]	2009 – 2010	ICU (1)	Mixed	Liberal definition: sepsis (two or more criteria transiently present during a 24 h period of automatic recording) plus refractory hypotension	Clinical data collected prospectively	All adult ICU admissions (not transferred or elective surgery)	At admission	Hospital: 23/51 (45.1)

					Restrictive definition: sepsis (≥ 3 criteria simultaneously present with manual recording at hourly intervals) plus refractory hypotension with other organ system failure	Clinical data collected prospectively	All adult ICU admissions (not transferred or elective surgery)	At admission	Hospital: 28/47 (59.6)
Denmark	Smith 2012 [65]	2009	ICU (6)	General	SEPSIS 1	Collected on paper case report forms and entered into an Excel data sheet	Consecutive ICU admissions with septic shock	Anytime during stay	30-day: 65/164 (39.6)
									90-day: 83/164 (50.6)
France	EPISS study (Pavon 2013 [46], Quenot 2013 [47])	2009 – 2011	ICU (14)	General	Infection requiring vasopressors despite adequate vascular filling with either metabolic acidosis, oliguria/renal insufficiency or hepatic dysfunction	Clinical data registered prospectively using standardized electronic case report forms	Consecutive adult ICU admissions	Anytime during stay	28-day: 625/1488 (42)
									ICU: 587/1488 (39.4)
									Hospital: 724/1488 (48.7)
USA	Miller 2013 [45]	2004 – 2010	ICUs (18)	Not reported	SEPSIS 2	Retrospective chart review and electronic health records	Consecutive ICU admissions	At admission	Hospital: 242/1426 (17)
France	Nesseler 2013 [69]	2008 – 2010	ICU (1)	Not reported	SEPSIS 1	Not reported	All patients with a first episode of septic shock that was present upon admission to the ICU or that developed during the course of ICU stay	Anytime during stay	ICU: 26/93 (28)
									Hospital: 30/93 (32.3)
									28-day: 24/93 (25.8)
									90-day: 37/93 (39.8)
									180-day: 42/93 (45.2)
Italy	Sakr 2013 [48]	2006	ICU (24)	Mixed medical/ surgical, surgical cardiac, neuro surgical	SEPSIS 1	Electronic case record forms entered into a multicenter regional database	All adult patients admitted to a network of 24 ICUs from 17 hospitals during a 180 day period	At admission	ICU: 56/100 (56)
								Anytime during stay	ICU: 85/145 (58.6)

Denmark	Storgaard 2013 [66]	2004 – 2007	Hospital (1)	Not reported	Infection and failure of ≥ 1 organ and systolic blood pressure ≤ 90 mmHg with no response to adequate volume therapy	Data extracted from 3 population-based registers: the Funen Patient Administrative System, the Danish National Registry of Patients, and the Civil Registration System	Patients hospitalized in one of the medical departments at Odense University Hospital	Anytime during stay	30-day: 44/103 (42.7)
									31 days - 4 years: 21/59 (35.6)
USA	Walkey 2013 [67]	2009	Hospital (up to 1000)	Not reported	SEPSIS 1	Discharge data from the Nationwide Inpatient Sample database	Admitted through the ED with suspected severe sepsis/septic shock	At admission	Hospital: NR/182,220 (31.4)
USA	Whittaker 2013 [68]	2005 – 2009	ED (1)	Not reported	SEPSIS 1/2	Case report forms	Patients admitted through the ED with a diagnosis of severe sepsis/septic shock	At admission	28-day: 117/321 (36.4)
Portugal	Goncalves-Pereira 2014 [50]	2009 – 2010	ICU (14)	Not reported	SEPSIS 1/2	Data collected prospectively into a multicenter database	Consecutive adult ICU admissions	At admission	Hospital: 418/856 (48.8)
USA	Lee 2014 [70]	2007 – 2009	ICU (1)	Medical	SEPSIS 2	Previously validated electronic medical database (ICU datamart)	Consecutive adult ICU admissions	Anytime during stay	Hospital: 142/594 (23.9)
Denmark	Rosland 2014 [71]	NR	ICU (7)	General	SEPSIS 1	Entered centrally in a local database from paper case report forms	Consecutive ICU admissions with septic shock	Anytime during stay	28-day: 102/213 (47.9)
									90-day: 114/213 (53.5)
France	Tolsma 2014 [51]	1997 – 2011	ICU (11)	Not reported	Not reported	OUTCOMEREA database generated from standardized report forms	All patients admitted to ICU OUTCOMEREA database	At admission	Day 28: 412/1094 (37.7)
France	Chauvet 2015 [52]	2008 – 2010	ICU (1)	Not reported	Not reported	Medical records and ICU observation charts	Consecutive ICU admissions	At admission	28-day: 83/218 (38.1)
									ICU: 66/218 (30.3)
Switzerland	Que 2015 [74]	2008 – 2012	ICU (1)	Mixed	SEPSIS 2	Electronic clinical report forms specifically designed for the study	Prospectively enrolled in the ICU with severe sepsis/septic shock	At admission	Hospital: 38/103 (36.9)

Europe (multiple)	Rhodes 2015 [73]	2013	ED/ICU (NR)	Not reported	SEPSIS 2	Clinical data registered prospectively using standardized electronic case report forms	Consecutive admissions to the ED or ICU with severe sepsis/septic shock	At admission to ED<br><br>Anytime during ICU stay	30-day (East Europe): 21/43 (48.8)<br>30-day (West Europe): 59/218 (27.1)
Spain	Suberviola Canas 2015 [72]	2005 – 2010	ICU (1)	Not reported	SEPSIS 2	Data collected prospectively	All adult ICU admissions with septic shock	At admission	ICU: 92/342 (26.9)<br>Hospital: 123/342 (36.0)
Finland	Ala-Kokko 2016 [75]	2011 – 2012	ICU (17)	Not reported	SEPSIS 1	Data collected prospectively into a multicenter national database	All adult ICU admissions (>24 hrs stay expected)	At admission	90-day: 61/178 (34.3)
Spain	Bouza 2016 [10]	2006 – 2001	Hospital (NR)	Not reported	Not reported	Data collected retrospectively from a national database	All hospitalized adult patients	Anytime during stay	Hospital: 50993/93380 (54.6)
USA	Bruns 2016 [76]	2009 – 2012	ED (1)	Not reported	Not reported	Retrospective chart review of a single center database	Consecutive ED admissions	At admission	Hospital: 72/166 (43.4)
France	Contou 2016 [77]	2014 – 2015	ICU (10)	Not reported	SEPSIS 1	Data collected prospectively	All consecutive ICU admissions with suspected septic shock	At admission	ICU (early confirmed septic shock): 139/374 (37.2)
USA	Drumheller 2016 [78]	2005 – 2009	ED (1)	Not reported	Arterial hypotension (systolic blood pressure <90 mm Hg) despite adequate fluid resuscitation (>	Data collected retrospectively from ED visit logs and electronic medical records	Patients admitted through the ED with severe sepsis and septic shock	Not reported	Hospital: 57/208 (27.4)

					1500mL) or use of vasopressors				
USA	Jones 2016 [79]	2012 – 2013	Hospital (6)	Not reported	Not reported	Data collected retrospectively from national databases	Hospitalized adult patients with a diagnosis of sepsis	Anytime during stay	Hospital: NR/1683 (36.4)
Italy	Mazzone 2016 [80]	2012	Hospital (31)	Not reported	SEPSIS 2	Data collected prospectively until hospital discharge	Consecutive hospitalized patients with an objective diagnosis of sepsis	At admission	Hospital: 2/17 (11.8)
Greece	Papadimitriou-Olivgeris 2016 [55]	2011 – 2014	ICU (1)	General	SEPSIS 3	Retrospective chart review of a single center database	All adult ICU admissions	Anytime during stay	ICU: 47/55 (85.5)
Germany	SepNet 2016 [54]	2013	ICU (133)	Not reported	SEPSIS 1&3	Data collected prospectively	All ICU admissions	At admission	Hospital mortality SEPSIS-1: 530/1224 (43.3) SEPSIS-3: 412/810 (50.9)
									ICU mortality SEPSIS-1: 473/1268 (37.3) SEPSIS-3: 371/838 (44.3)
USA	Adams 2017 [81]	2010 – 2014	ICU (2)	Not reported	Per physician diagnosis	Retrospective chart review	Consecutive adult ICU admissions	Anytime during stay	All-cause: 160/308 (51.9)
Spain and France	Andaluz-Ojeda 2017 [82]	2013 – 2016	ICU (2)	Not reported	SEPSIS 2	Data collected prospectively	Consecutive adult ICU admissions with severe sepsis/septic shock	Anytime during stay	28-day: 87/239 (36.4)

USA	Beesley 2017 [83]	2006 – 2013	ICU (3)	Not reported	SEPSIS 1 (modified)	Review of medical records	Adult ICU admissions with septic shock requiring vasopressor therapy	At admission	28-day: 435/1554 (28.0)
USA	Henning 2017 [84]	2012 – 2013	ED (1)	Not reported	Systolic blood pressure <90 mm Hg after ≥ 1L IV fluids, new vasopressor requirement, or systolic blood pressure <90 mm Hg and IV fluids held for concern of fluid overload	Institutional database and medical records	All consecutive adult ED admissions with suspected infection	At admission	Hospital: 79/378 (20.9)
France	Jamme 2017 [85]	2008 – 2015	ICU (1)	Medical	SEPSIS 2	Retrospective chart review	All adult ICU admissions with septic shock diagnosed within 48 hrs	At admission (48 hours)	3-day: 113/801 (14.1)<br>ICU: 299/801 (37.3)<br>Hospital: 331/801 (41.3)
USA	Kadri 2017 [86]	2005 – 2014	Hospital (27)	Not reported	SEPSIS 1/2	Retrospective chart review of a multicenter national database	All hospitalized adult patients	Anytime during stay	Hospital mortality Septic Shock Surveillance Definition: 51,507/99,312 (51.9)<br>Hospital mortality ICD-9 Code for Septic Shock: 34,665/82,350 (42.1)
USA	Liu 2017 [87]	2010 – 2013	ED (21)	Not reported	SEPSIS 2 (modified)	Data collected retrospectively from national databases	Adult patients hospitalized through the ED with a diagnosis of sepsis	At admission	Hospital:1215/4,668 (26.0)
USA	Marik 2017 [88]	2013	ICU (500)	Not reported	Not reported	Retrospective chart review of a multicenter national database	All adult non-surgical ICU patients admitted from the ED with severe sepsis/septic shock and parenteral antibiotics on day 1	At admission	Hospital: NR/14531 (32.6)

Netherlands	Ong 2017 [89]	2011 – 2014	ICU (2)	Mixed	Presence of sepsis plus the use of noradrenaline for hypotension in a dose of >0.1 μg/kg/min for more than 12 hours during the first 3 days in the ICU	Data collected prospectively	Consecutive adult ICU admissions with septic shock	At admission	ICU: 81/329 (24.6)
USA	Saqib 2017 [90]	2003 – 2011	Hospital (NR)	Not reported	Not reported	Data collected retrospectively from a multicenter national database	Hospitalized patients with septic shock	At admission	Hospital mortality May: NR/118808 (39.6)<br>Hospital mortality July: NR /119798 (38.6)
USA	Schuetz 2017 [91]	2011 – 2014	ED/ICU (13)	Not reported	SIRS Criteria I + II and state of acute circulatory failure characterized by persistent arterial hypotension unexplained by other causes	Data collected prospectively	Adult patients with severe sepsis/septic shock treated in the ICU or admitted from the ED/other wards	At admission	28-day all-cause: 54/299 (18.1)
UK	Shankar-Hari 2017 [57]	2011 – 2015	ICU (189)	Adult general	SEPSIS 2&3	Data collected prospectively into a multicenter national database	Consecutive adult ICU admissions	At admission	ICU mortality SEPSIS 2: 39294/153257 (25.6) SEPSIS 3: 18338/39262 (46.7)<br>Hospital mortality SEPSIS 2: 49656/153257 (32.4) SEPSIS 3: 20457/39262 (52.1)

ACCP/SCCM, American College of Chest Physicians/Society of Critical Care Medicine; BSI, bloodstream infection; ED, emergency department; EPISS, EPIdemiology of Septic Shock study; ICD, International classification of Diseases; ICU, intensive care unit; NR, not reported; SACiUCI, Portuguese Community-Acquired Sepsis study; SIRS, systemic inflammatory response syndrome; US, United States of America; SOAP, Sepsis Occurrence in Acutely Ill Patients study

Table S3. Quality assessment of frequency studies using the Joanna Briggs Institute tool

Question	Yes (%)	No (%)	Unclear (%)
Was the sample frame appropriate to address the target population?	26 (74.3)	6 (17.1)	3 (8.6)
Were study participants sampled in an appropriate way?	30 (85.7)	1 (2.9)	4 (11.4)
Was the sample size adequate?	35 (100)	0	0
Were the study subjects and the setting described in detail?	26 (74.3)	8 (22.9)	1 (2.9)
Was the data analysis conducted with sufficient coverage of the identified sample?	33 (94.3)	0	1 (5.7)
Were valid methods used for the identification of the condition?	25 (71.4)	4 (11.4)	5 (17.1)
Was the condition measured in a standard, reliable way for all participants?	21 (60)	1 (2.9)	13 (37.1)
Was there appropriate statistical analysis, i.e. in relation to calculating frequency?	27 (77.1)	4 (11.4)	4 (11.4)
Was the response rate adequate, and if not, was the low response rate managed appropriately?	25 (71.4)	0	9 (28.6)

Table S4. Quality assessment of mortality studies using a modified Joanna Briggs Institute tool for the assessment of case series

Question	Yes (%)	No (%)	Unclear (%)
Were there clear criteria for inclusion in the case series?	54 (94.7)	3 (5.3)	0
Was the condition measured in a standard, reliable way for all septic shock patients included in the case series?	33 (57.9)	2 (3.5)	22 (37.9)
Were valid methods used for the identification of the condition for all septic shock patients included in the case series?	35 (61.4)	7 (12.3)	15 (25.9)
Did the case series have consecutive inclusion of septic shock patients?	43 (75.4)	8 (14.0)	6 (10.3)
Did the case series have complete inclusion of septic shock patients?	28 (49.1)	22 (38.6)	7 (12.1)
Was there clear reporting of the demographics of the septic shock patients in the study?	32 (56.1)	25 (43.9)	0 (0)
Was there clear reporting of the clinical information of the septic shock patients in the study?	36 (63.2)	21 (36.8)	0 (0)
Was the mortality of cases clearly reported?	49 (86.0)	8 (14.0)	0 (0)
Was there clear reporting of the presenting site(s)/clinic(s) demographic information?	33 (57.9)	20 (35.1)	4 (6.9)
Was statistical analysis appropriate to calculate septic shock mortality?	50 (87.7)	6 (10.5)	1 (1.7)

Figure S2. Random effects meta-analysis of studies frequency of septic shock in European (panel a) and North American (panel b) cohorts of patients admitted to the intensive care unit and diagnosed at admission

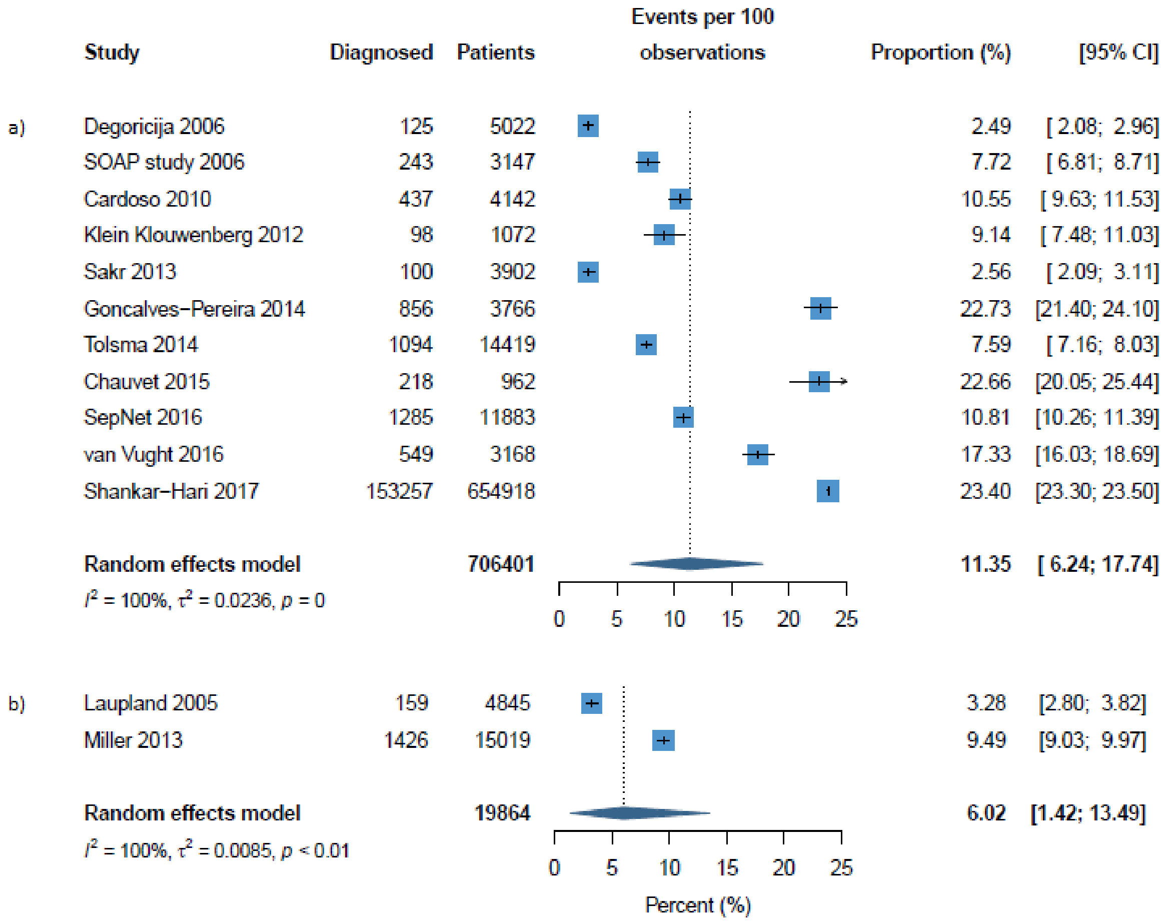

The forest plots contain exact 95% confidence intervals, and specific studies are weighted using the inverse-variance method. The pooled summaries are obtained using the Freeman–Tukey double arcsine transformation and the DerSimonian–Laird method estimates between-study variance.

Figure S3. Random effects meta-analysis of studies reporting frequency of septic shock in European (panel a) and North American (panel b) cohorts of patients admitted to the intensive care unit and diagnosed at any time during the ICU stay

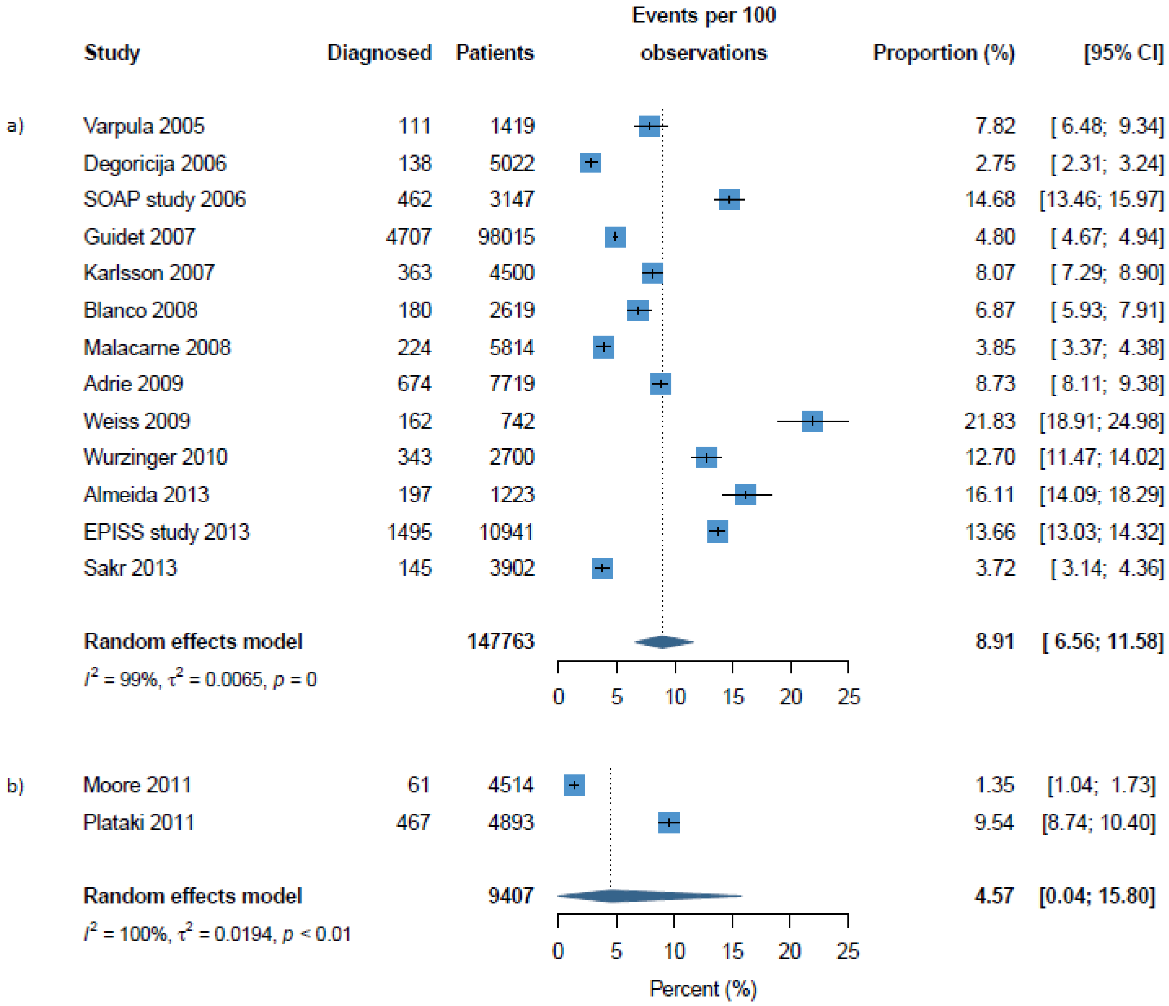

The forest plots contain exact 95% confidence intervals, and specific studies are weighted using the inverse-variance method. The pooled summaries are obtained using the Freeman–Tukey double arcsine transformation and the DerSimonian–Laird method estimates between-study variance.

Figure S4. Random effects meta-analysis of single center (panel a) and multicenter (panel b) studies reporting frequency of septic shock on admission to the intensive care unit

a)

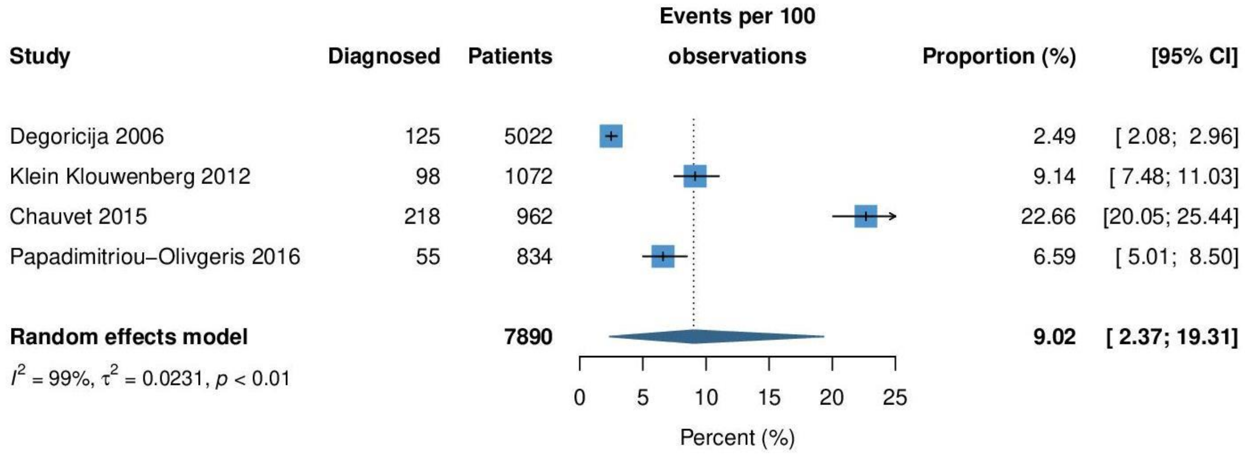

b)

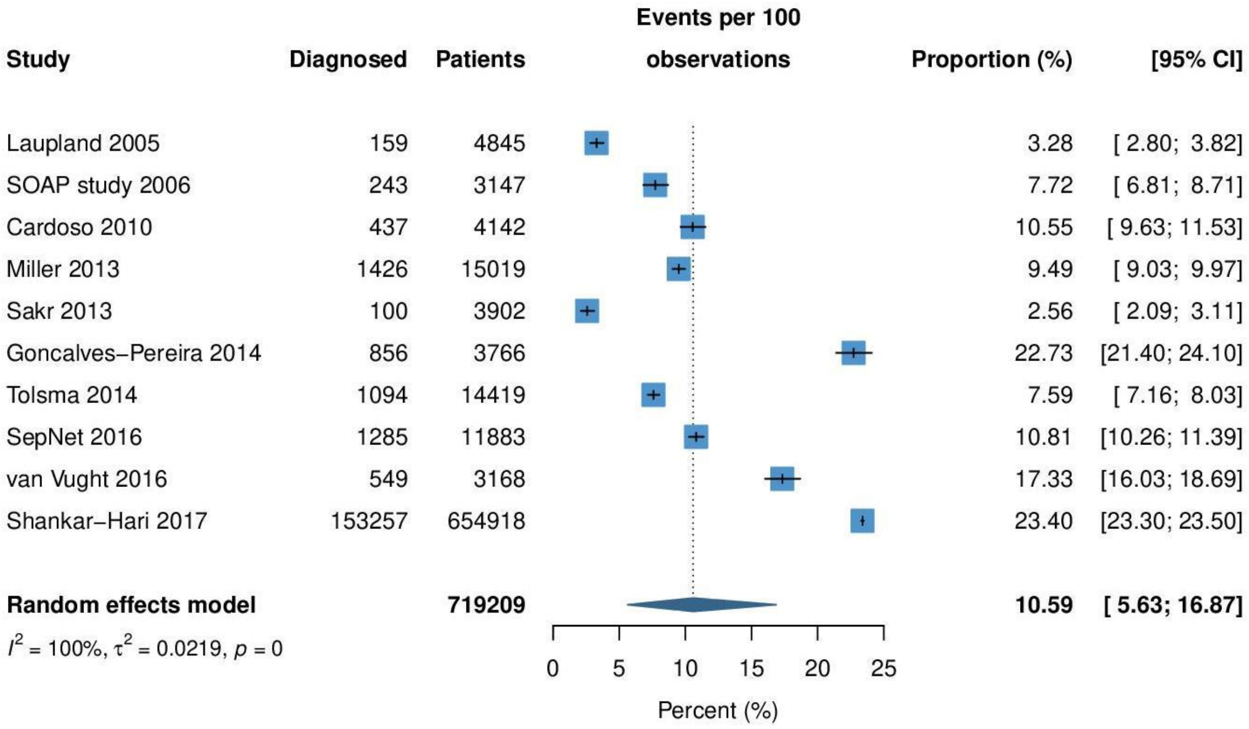

The forest plots contain exact 95% confidence intervals, and specific studies are weighted using the inverse-variance method. The pooled summaries are obtained using the Freeman–Tukey double arcsine transformation and the DerSimonian–Laird method estimates between-study variance.

Figure S5. Random effects meta-analysis of single center (panel a) and multicenter (panel b) studies reporting frequency of septic shock diagnosed at any time during the ICU stay

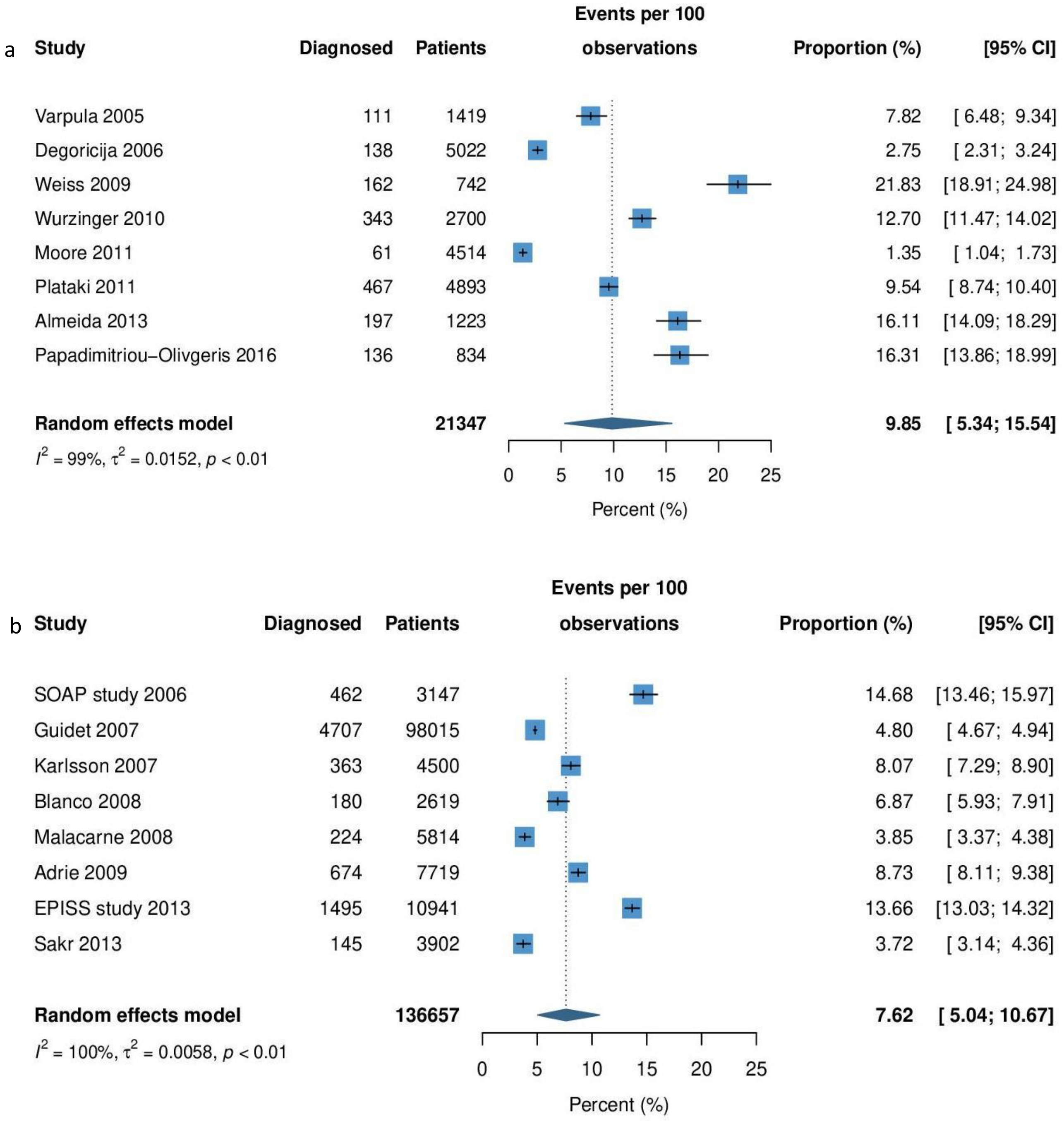

The forest plots contain exact 95% confidence intervals, and specific studies are weighted using the inverse-variance method. The pooled summaries are obtained using the Freeman–Tukey double arcsine transformation and the DerSimonian–Laird method estimates between-study variance.

Figure S6. Random effects meta-analysis of studies reporting ICU mortality of septic shock patients in Europe (panel a) and North America (panel b)

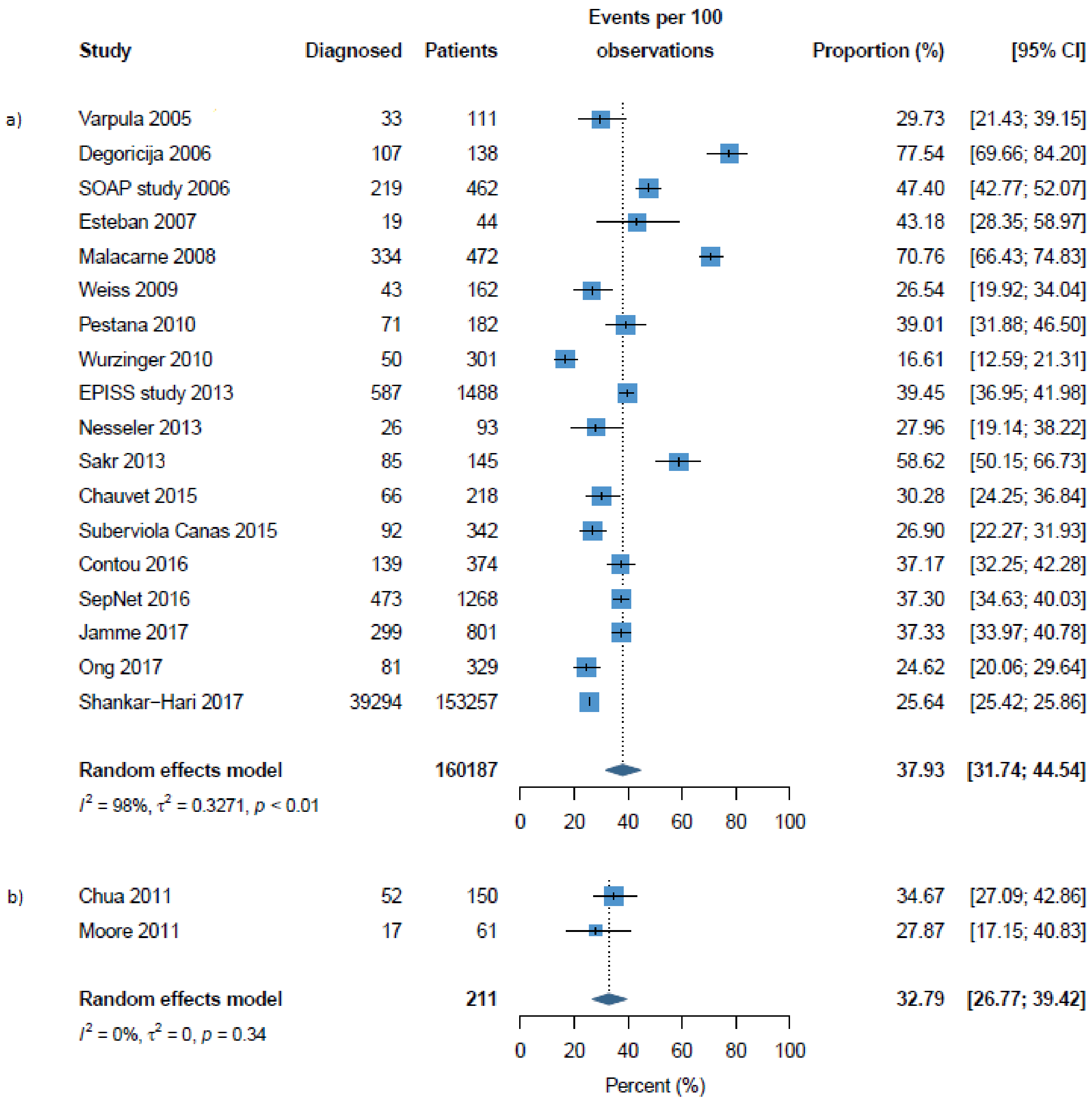

The forest plots contain exact 95% confidence intervals, and specific studies are weighted using the inverse-variance method. The pooled summaries are obtained using the logit transformation and the DerSimonian–Laird method estimates between-study variance.

Figure S7. Random effects meta-analysis of studies reporting hospital mortality of septic shock patients in Europe (panel a) and North America (panel b)

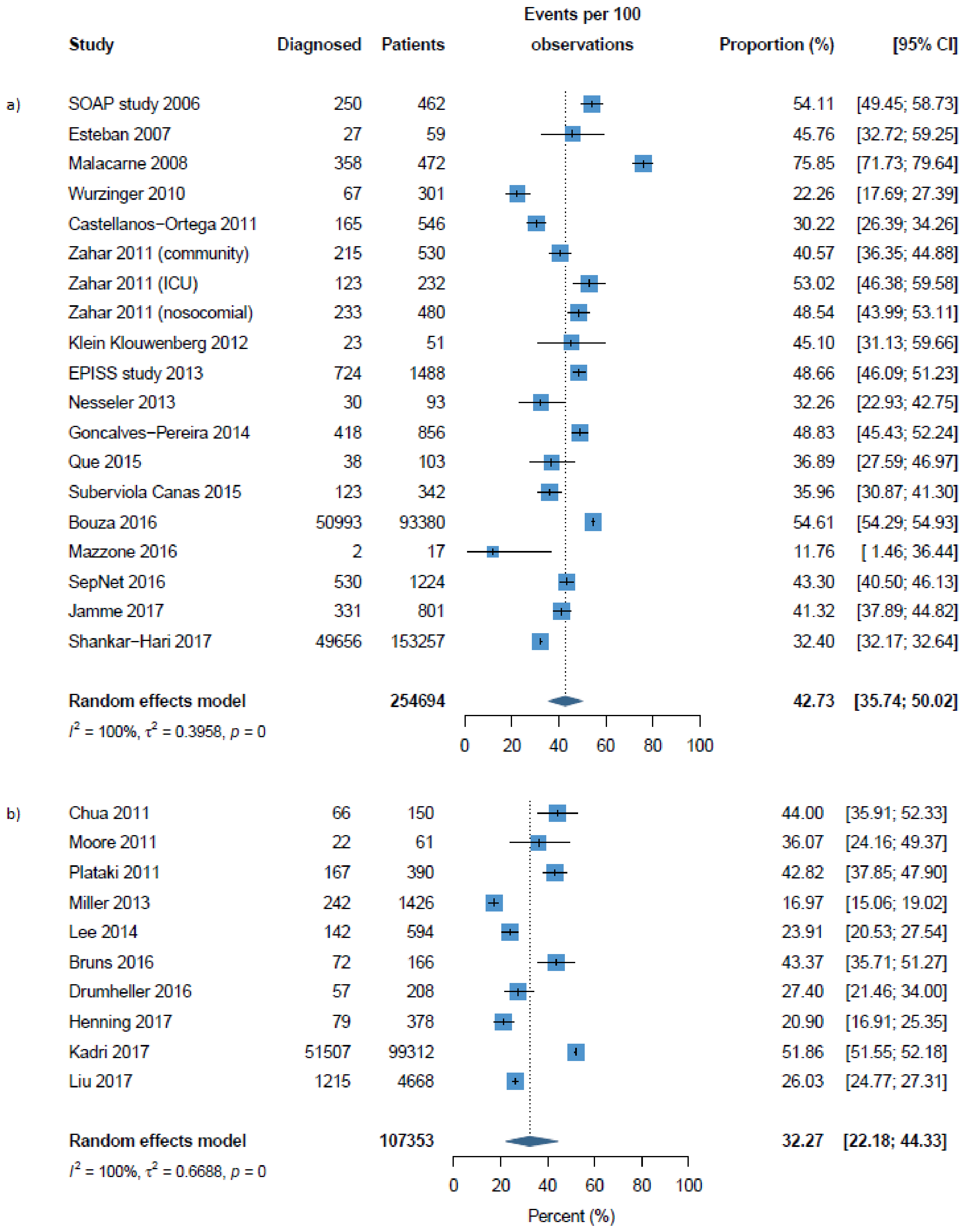

The forest plots contain exact 95% confidence intervals, and specific studies are weighted using the inverse-variance method. The pooled summaries are obtained using the logit transformation and the DerSimonian–Laird method estimates between-study variance.

Figure S8. Random effects meta-analysis of studies reporting mortality at 28 / 30 days in septic shock patients in Europe (panel a) and North America (panel b)

	Study	Diagnosed	Patients	Events per 100 observations	Proportion (%)	[95% CI]
a)	Varpula 2005	36	111		32.43	[23.85; 41.97]
	SACiUCI study 2009	191	437		43.71	[39.00; 48.50]
	Smith 2012	65	164		39.63	[32.09; 47.56]
	EPISS study 2013	625	1488		42.00	[39.48; 44.56]
	Nesseler 2013	24	93		25.81	[17.29; 35.92]
	Storgaard 2013	44	103		42.72	[33.02; 52.85]
	Rosland 2014	102	213		47.89	[41.01; 54.82]
	Tolsma 2014	412	1094		37.66	[34.78; 40.61]
	Chauvet 2015	83	218		38.07	[31.60; 44.88]
	Rhodes 2015 (E. Europe)	21	43		48.84	[33.31; 64.54]
	Rhodes 2015 (W. Europe)	59	218		27.06	[21.29; 33.48]
	Andaluz–Ojeda 2017	87	239		36.40	[30.30; 42.85]
	**Random effects model**		**4421**		**38.54**	**[35.36; 41.82]**
	$I^2 = 73\%$, $\tau^2 = 0.0360$, $p < 0.01$					

	Study	Diagnosed	Patients	Events per 100 observations	Proportion (%)	[95% CI]
b)	Laupland 2005	81	159		50.94	[42.91; 58.95]
	Mikkelsen 2009	74	196		37.76	[30.95; 44.94]
	Whittaker 2013	117	321		36.45	[31.17; 41.98]
	Beesley 2017	435	1554		27.99	[25.77; 30.30]
	Schuetz 2017	54	299		18.06	[13.87; 22.90]
	**Random effects model**		**2529**		**33.18**	**[24.82; 42.75]**
	$I^2 = 94\%$, $\tau^2 = 0.1998$, $p < 0.01$					

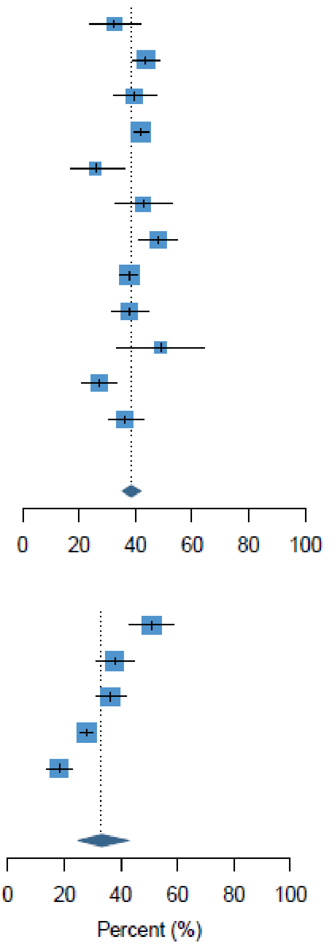

The forest plots contain exact 95% confidence intervals, and specific studies are weighted using the inverse-variance method. The pooled summaries are obtained using the logit transformation and the DerSimonian–Laird method estimates between-study variance.

Figure S9. Random effects meta-analysis of single center (panel a) and multicenter (panel b) studies reporting ICU mortality in septic shock patients

a

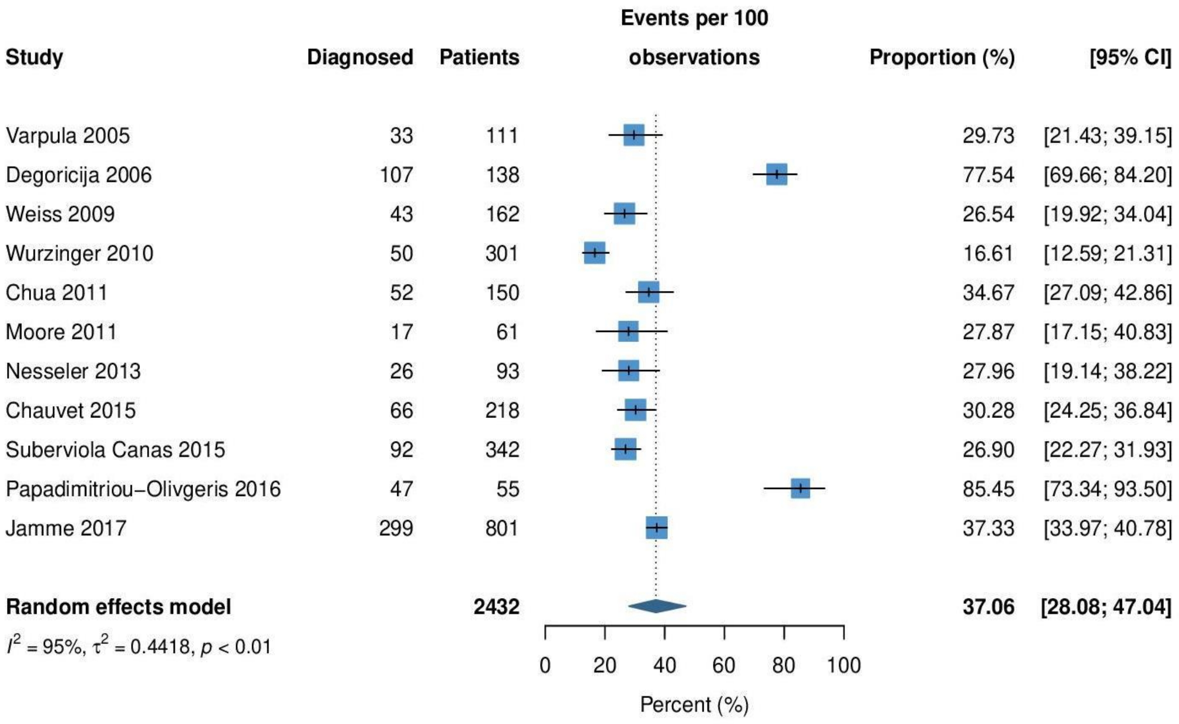

b

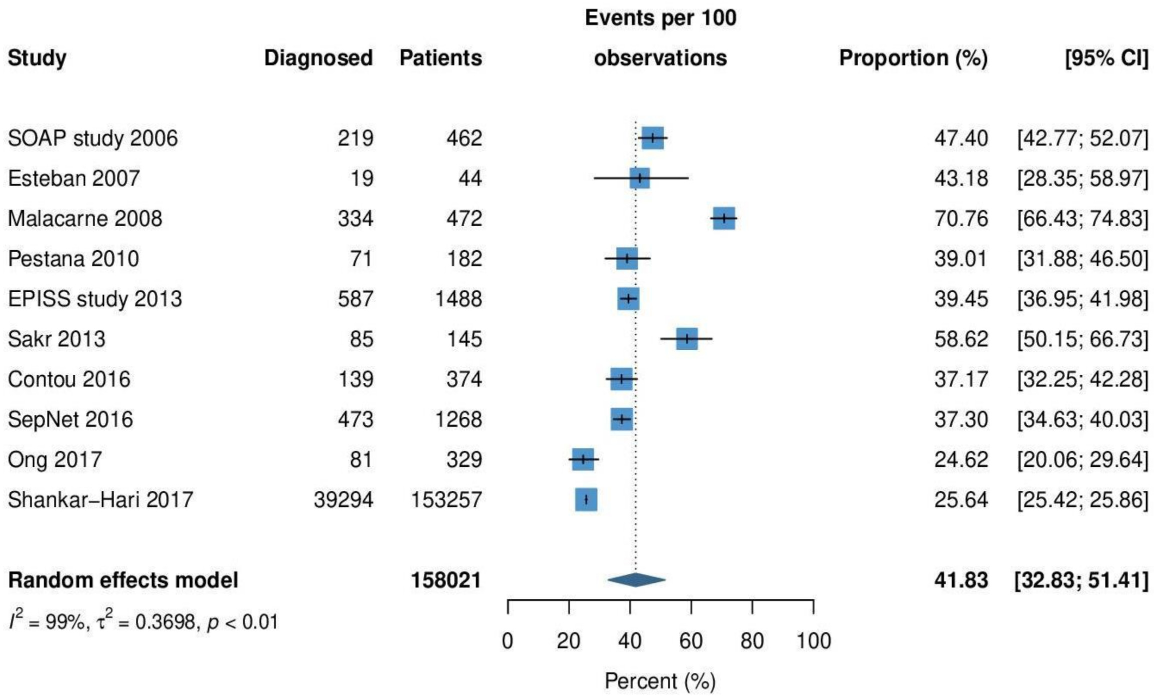

The forest plots contain exact 95% confidence intervals, and specific studies are weighted using the inverse-variance method. The pooled summaries are obtained using the logit transformation and the DerSimonian–Laird method estimates between-study variance.

Figure S10. Random effects meta-analysis of single center (panel a) and multicenter (panel b) studies reporting hospital mortality in septic shock patients

a

Study	Diagnosed	Patients	Proportion (%)	[95% CI]
Wurzinger 2010	67	301	22.26	[17.69; 27.39]
Chua 2011	66	150	44.00	[35.91; 52.33]
Moore 2011	22	61	36.07	[24.16; 49.37]
Plataki 2011	167	390	42.82	[37.85; 47.90]
Klein Klouwenberg 2012	23	51	45.10	[31.13; 59.66]
Nesseler 2013	30	93	32.26	[22.93; 42.75]
Lee 2014	142	594	23.91	[20.53; 27.54]
Que 2015	38	103	36.89	[27.59; 46.97]
Suberviola Canas 2015	123	342	35.96	[30.87; 41.30]
Bruns 2016	72	166	43.37	[35.71; 51.27]
Drumheller 2016	57	208	27.40	[21.46; 34.00]
Henning 2017	79	378	20.90	[16.91; 25.35]
Jamme 2017	331	801	41.32	[37.89; 44.82]
**Random effects model**		**3638**	**33.98**	**[28.83; 39.54]**

$I^2 = 90\%$, $\tau^2 = 0.1665$, $p < 0.01$

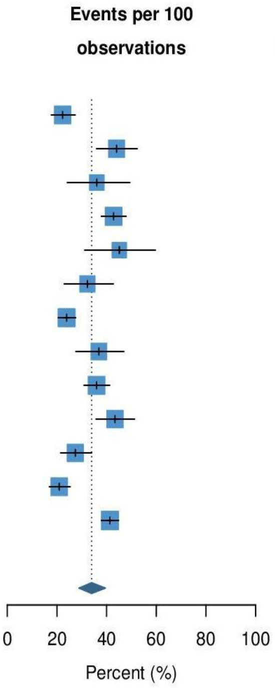

b

Study	Diagnosed	Patients	Proportion (%)	[95% CI]
SOAP study 2006	250	462	54.11	[49.45; 58.73]
Esteban 2007	27	59	45.76	[32.72; 59.25]
Malacarne 2008	358	472	75.85	[71.73; 79.64]
Castellanos–Ortega 2011	165	546	30.22	[26.39; 34.26]
Zahar 2011 (community)	215	530	40.57	[36.35; 44.88]
Zahar 2011 (ICU)	123	232	53.02	[46.38; 59.58]
Zahar 2011 (nosocomial)	233	480	48.54	[43.99; 53.11]
EPISS study 2013	724	1488	48.66	[46.09; 51.23]
Miller 2013	242	1426	16.97	[15.06; 19.02]
Goncalves–Pereira 2014	418	856	48.83	[45.43; 52.24]
Bouza 2016	50993	93380	54.61	[54.29; 54.93]
Mazzone 2016	2	17	11.76	[1.46; 36.44]
SepNet 2016	530	1224	43.30	[40.50; 46.13]
Kadri 2017	51507	99312	51.86	[51.55; 52.18]
Liu 2017	1215	4668	26.03	[24.77; 27.31]
Shankar–Hari 2017	49656	153257	32.40	[32.17; 32.64]
**Random effects model**		**358409**	**43.12**	**[36.66; 49.82]**

$I^2 = 100\%$, $\tau^2 = 0.2816$, $p = 0$

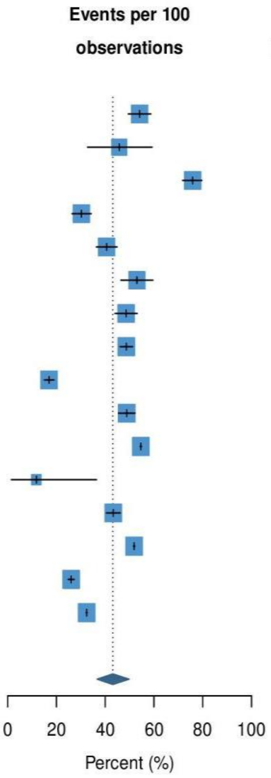

The forest plots contain exact 95% confidence intervals, and specific studies are weighted using the inverse-variance method. The pooled summaries are obtained using the logit transformation and the DerSimonian–Laird method estimates between-study variance.

Figure S11. Random effects meta-analysis of single center (panel a) and multicenter (panel b) studies reporting 28/30-day mortality in septic shock patients

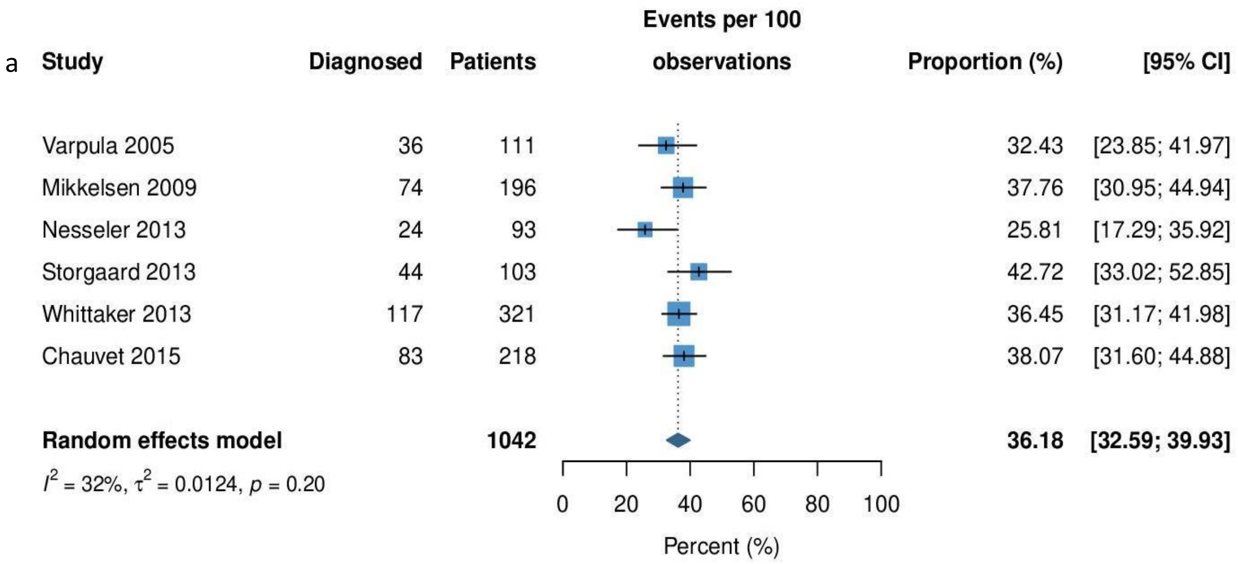

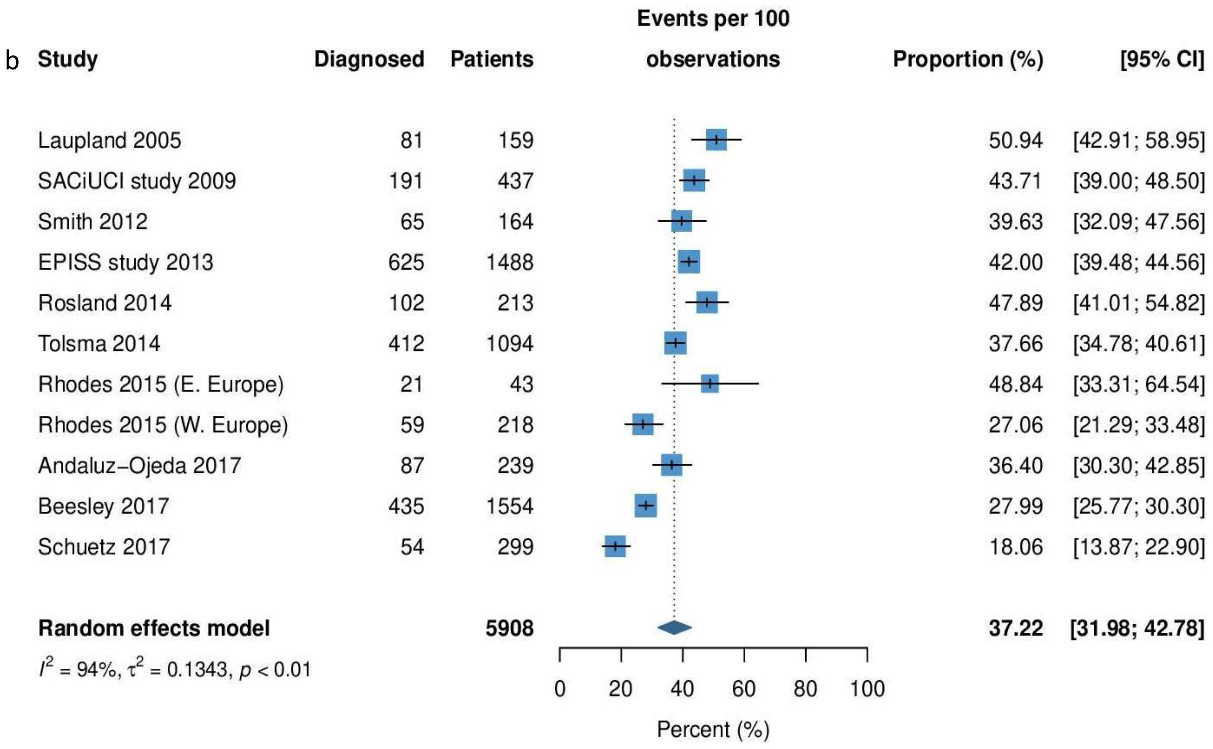

The forest plots contain exact 95% confidence intervals, and specific studies are weighted using the inverse-variance method. The pooled summaries are obtained using the logit transformation and the DerSimonian–Laird method estimates between-study variance.
